# An Exploratory Study on the Regulatory Effect of Autonomous Sensory Meridian Response on Anxiety: Evidence From Functional Near‐Infrared Brain Imaging Technology

**DOI:** 10.1111/ejn.70251

**Published:** 2025-09-14

**Authors:** Huan Jiang, Yating Chen, Feng Guo, Binjie Yang, Jiani Yan, HuiLing Li, Ke Jiang, Qiang Zhou, Xianghe Zhu

**Affiliations:** ^1^ Department of psychology Wenzhou Medical University Wenzhou China; ^2^ Shanghai Key Laboratory of Brain Functional Genomics (Ministry of Education), School of Psychology and Cognitive Science East China Normal University Shanghai China; ^3^ Lishui Second Hospital Affiliated to Wenzhou Medical University Lishui China; ^4^ Zhejiang Provincial Clinical Research Center for Mental Disorders, The Affiliated Kangning Hospital, Wenzhou Medical University Wenzhou China; ^5^ Institute of Aging, Key Laboratory of Alzheimer's Disease of Zhejiang Province Wenzhou Medical University Wenzhou China; ^6^ Institute of Wenzhou Zhejiang University Wenzhou China

**Keywords:** ASMR, fNIRS, state anxiety, trait anxiety

## Abstract

While some studies have suggested that autonomous sensory meridian response (ASMR) can relieve anxiety, whether ASMR relieves anxiety in trait‐anxious individuals and its neural mechanisms remain a question worth exploring. We used the self‐threatening *n*‐back paradigm to elicit state anxiety and an ASMR video to relieve anxiety. Functional near‐infrared spectroscopy (fNIRS) was used to monitor frontal and temporal lobe activity to explore the neurophysiological changes when watching ASMR videos and then further explore the mechanism of self‐reported “feelings” of ASMR. During the anxiety arousal phase, the hemodynamic responses (HR) of the middle frontal gyrus (MFG) and prefrontal cortex (PFC) were significantly enhanced in participants with high trait anxiety. Those with low‐trait anxiety had significantly reduced HR of the dorsolateral prefrontal cortex (DLPFC). Compared with the non‐ASMR video, the HR of the MFG, PFC, and DLPFC were significantly lower in participants who watched the ASMR video. When the experience of ASMR was reported, the HR of the premotor and auxiliary motor cortices, middle frontal lobe, and DLPFC were significantly reduced. While watching an ASMR video, brain activity is the opposite of when anxiety is elicited in people with high trait anxiety. The activated area when the participants were watching the ASMR video, and when they reported experiencing ASMR, overlapped with the anxiety‐related parts of the brain. This provides neurophysiological evidence of how an ASMR video can relieve anxiety.

## Graphical abstract

We employed the self‐threatening *n*‐back paradigm to induce participants' state anxiety. Following this induction, participants were exposed to an Autonomous Sensory Meridian Response (ASMR) video, which purportedly aids in anxiety alleviation. Throughout the anxiety arousal phase, participants exhibited heightened neural activity within anxiety‐related brain regions, particularly among those with elevated trait anxiety levels. Subsequent to viewing the ASMR video, a reduction in neural activity within these regions was observed. This neurophysiological response furnishes evidence supporting the anxiety‐relieving potential of ASMR videos.

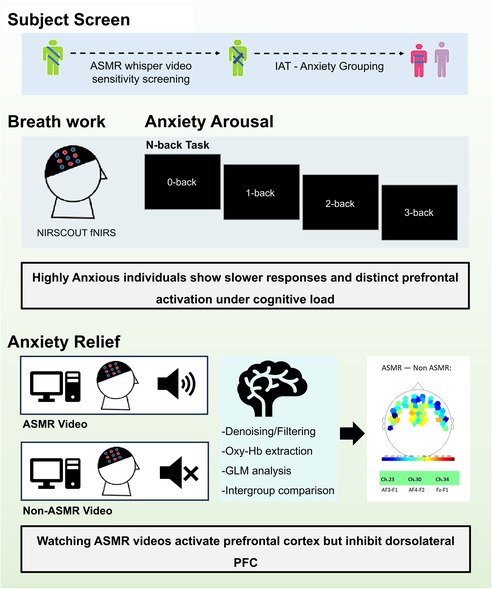

**Abbreviations**
ASMR: autonomous sensory meridian response
DLPFC: dorsolateral prefrontal cortex
DMN: default mode network
fMRI: functional magnetic resonance imaging
fNIRS: functional near‐infrared spectroscopy
HR: hemodynamic responses
MFG: middle frontal gyrus
PFC: prefrontal cortex

## 1 Introduction

Anxiety—an organic response that triggers worry and heightens alertness in the face of uncertainty, danger, or potential threats to well‐being—profoundly impacts individuals' lives (Huang and Ren 2013). While moderate anxiety can promote progress, excessive anxiety harms both individuals and society; lack of proper relief may lead to anxiety disorders. Current treatments for anxiety disorders primarily include psychological interventions and pharmacotherapy. A meta‐analysis has revealed that pharmacotherapy tends to be more efficacious than psychotherapy (Bandelow et al. 2015). However, many patients remain inclined to accept psychological interventions due to the fear of side effects or the difficulties of taking medications. Regardless of the treatment modality, only a fraction—approximately 20% of young people diagnosed with anxiety achieve sustained well‐being (Leal et al. 2017). Convenient self‐help methods for relieving anxiety can help to prevent it before it occurs. With the advancement of the Internet, some novel techniques have emerged, but their efficacy requires further validation at the neurophysiological level.

Audiovisual materials can elicit positive experiences, thereby alleviating negative emotional states. As early as 1998, Fredrickson and Levenson found that watching positive movies promoted better recovery of positive emotions and more rapid return to prefilm levels of cardiovascular activation compared to neutral and negative films. In the contemporary digital landscape, autonomous sensory meridian response (ASMR) creators on YouTube incorporate visual elements, sounds, motifs of touching and tasting, and other scenarios in multisensory video interactions to deliver enjoyable and relaxing experiences to their viewers (Niu et al. 2022). ASMR, an acronym coined in 2010 to denote the profound relaxation often accompanied by a pleasurable tingling sensation on the scalp, is considered an internet phenomenon with potential anxiety‐reducing properties. An exploratory study by Barratt and Davis (2015) revealed that nearly 80% of ASMR participants reported that ASMR had a positive mood‐altering effect, and that ASMR stimulation helped them relax, sleep, and reduce anxiety (Barratt et al. 2017; Cash et al. 2018; Poerio et al. 2018). Audiences use these videos to elicit ASMR, enhancing relaxation and sleep quality; such videos even serve as a coping mechanism for depression and anxiety relief. Smejka and Wiggs (2022) found that all participants showed significant increments in relaxation and mood improvement, with those who experienced ASMR showing the most pronounced effects. Participants have reported that the whispering state generates the most frequent, strongest tingling sensations; increases positive emotions and behaviors (namely tranquility); produces less anxiety; and fosters the ability to connect with others (Barratt and Davis 2015; Scofield 2019).

What, then, are the neurophysiological alterations related to ASMR experience? A growing body of research is dedicated to exploring the brain mechanism of individuals in a baseline state under the regulation of ASMR. Neuroimaging studies into functional connectivity and default mode network (DMN) have shown that ASMR participants enhanced connectivity within the frontal and temporal lobes, as well as in sensory and attention‐related regions, when compared to control subjects (Lee et al. 2020; Smith et al. 2017; Smith et al. 2019b). In task‐based functional magnetic resonance imaging (fMRI) studies, Lochte et al. (2018) reported neural activity during ASMR‐induced tingling sensation; participants showed significant activation in regions associated with both reward and emotional arousal (the nucleus accumbens, dorsal anterior cingulate cortex, insular cortex, and inferior frontal gyrus). Similarly, Smith et al. (2019a) also found ASMR videos elicit activity in brain areas related to sensation, emotion, and attention. Electroencephalography (EEG) studies, on the other hand, have found that ASMR stimulation elicited increases in alpha waves, gamma waves, and sensorimotor rhythms, suggesting the influence of ASMR in attentional and sensorimotor features (Fredborg et al. 2021; Seifzadeh et al. 2021).

Anxiety is highly correlated with central executive functions involving attentional control, which subsequently impacts the efficiency of task processing (Eysenck et al. 2007), and the relief of “overload” in anxious participants leads to dilution of cognitive resources, especially affecting processes related to inhibition and attention shift (Berggren and Derakshan 2013), which can be observed when cognitive load is low (Berggren et al. 2013). Studies examining these phenomena have mainly focused on the relationship between the anxiety level and working memory or the perceptual load of the frontal lobe, especially the prefrontal lobe (Basten et al. 2012; Bishop 2009).

Studies of the neurophysiological mechanisms of anxiety used the *n*‐back paradigm to elicit state anxiety frequently. A meta‐analysis has revealed that anxiety has the capacity to degrade performance on the *n*‐back task, a standard measure of working memory capacity (Moran 2016). Furthermore, anxiety may affect the executive part of working memory (Eysenck and Derakshan 2011). A study of verbal and spatial *n*‐back tasks involving psychological matching also showed that induced anxiety impairs performance on the spatial *n*‐back task (Lavric et al. 2003). The load level of the *n*‐back task is adjustable parametrically, and the verbal and spatial versions of the *n*‐back exhibit comparable psychometric properties (Shackman et al. 2006). Empirical evidence has indicated that with the facial expressions of the *n*‐back task, there is a positive correlation between negative emotions in the medial prefrontal cortex and hemodynamic responses, indicating that external stimuli can modulate participants' emotional states (Ozawa et al. 2014). Moreover, there is a negative correlation between state anxiety and hemodynamic responses in the right hemisphere, and the right lateralization of the prefrontal lobe is negatively correlated with the level of state anxiety (Tseng et al. 2018). Consequently, state anxiety can be induced and indirectly assessed through cognitive tasks (e.g., the *n*‐back experimental paradigm).

The neural mechanisms by which ASMR alleviates anxiety are not yet fully explored. Both fMRI and functional near‐infrared spectroscopy (fNIRS) specialize in detecting spatial differences within the brain, demonstrating high correlation and reliability (Noah et al. 2015). Compared to fMRI, fNIRS offers a more open and ecologically valid testing environment, compensates for the limitations of closed experimental environments, and better captures changes in brain tissue during daily activities. In ASMR studies, differences between laboratory and natural environments may affect the ecological validity of the study. ASMR is often experienced in comfortable, familiar environments, which may not be fully replicated in labs, affecting responses (Poerio et al. 2018). For highly anxious participants, lab settings might increase discomfort. Therefore, to better understand the neurophysiological mechanisms of ASMR in daily life and to minimize the gap between the lab and natural settings, we will use fNIRS to explore the neural changes during ASMR video viewing in an anxious state. A self‐reported “sensation” button will be included to further investigate the subjective ASMR experience.

In addition, the effectiveness of ASMR in relieving anxiety may also be influenced by the individual's traits. Anxiety is divided into state and trait anxiety. State anxiety reflects the temporary, participative fear of potential threats or negative experiences (Pine and LeDoux 2017); trait anxiety is conceptualized as a personality trait, describing and presenting individual differences related to the current tendencies of state anxiety (Leal et al. 2017). Trait anxiety is related to the internal connection of the cortical midline structures, whereas state anxiety is more closely tied to the functional dynamics of the insula (Tian et al. 2016). A shared neural substrate underlies both state and trait anxiety, indicating a functional and structural interconnection between these two constructs (Baur et al. 2013). These findings underline the distinct yet shared mechanisms underlying state and trait anxiety. The two are also closely connected in terms of behavioral performance. The higher the trait anxiety, the higher the state anxiety in light of different threats (Beato et al. 2013). The ventromedial prefrontal cortex is also linked to state anxiety in response to threats (Hu 2018). Hence, the impact of threat‐related scenarios on state anxiety varies among individuals with different levels of trait anxiety. As trait anxiety affects changes in state anxiety across contexts (Leal et al. 2017; Tian et al. 2016), taking trait anxiety into account when examining changes in state anxiety across experimental conditions would make the results more accurate. Past research has focused only on state anxiety after arousal when examining the physiological mechanisms of anxiety, ignoring the influence of individual trait anxiety (Ozawa et al. 2014; Tseng et al. 2018). Our combined study of trait anxiety and state anxiety will creatively break through the logical dilemma of individual differences and temporal dimensions of anxious populations in previous related studies. The IAT‐Anxiety is a reliable measure to predict criterion variables above questionnaire measures of anxiety, and its validity is not compromised by fake responses (Egloff and Schmukle 2002), preventing the participants from guessing the purpose of the experiment before the experiment begins.

As previously reviewed, most of the research on ASMR for anxiety relief has focused on task‐induced state anxiety, and few studies have been conducted on trait anxiety. Eid et al. (2022) found that participants' levels of state anxiety decreased significantly after viewing ASMR videos, highlighting the significant mediating role of personality traits, including trait anxiety and neuroticism, in this anxiety reduction. Therefore, the effect of trait anxiety should be considered when investigating the mechanism of action of ASMR on anxiety. In sum, we used fNIRS to monitor cerebral blood oxygen changes in the frontotemporal lobes when individuals with high‐ and low‐trait anxiety experienced ASMR in an anxiety state. We also sought to have further understanding of the therapeutic mechanism of ASMR in regulating anxiety.

## 2 Method

### 2.1 Research Design

We used a two‐factor mixed design; the between‐subjects variable was trait anxiety (high‐trait anxiety/low‐trait anxiety) and the within‐subjects variable was the type of video watched (ASMR/non‐ASMR).

Our study was approved by an ethics committee, the ethical lot number is 2020‐006, and the approval unit is the Ethics Committee of Wenzhou Medical University. All participants voluntarily took part and signed an informed consent form; they could withdraw at any time if they were unwilling or unable to continue with the experiment.

### 2.2 Participants

For the behavioral data, the number of participants required was 34 by G‐power, and the “ANOVA: Repeated measures, within‐between interaction” was used as a statistical test, with the relevant parameters set as effect size *f* = 0.25, power(1‐β) = 0.8; for the fNIRS data, the required number of participants was 12 by G‐power and the “Means: Difference between two dependent means (matched pairs)” test was used, with the relevant parameters set to effect size d_z_ = 0.8 (Smith et al. 2019a, 2019b), power(1‐β) = 0.8.

We screened 395 college students, excluding those with invalid data due to incomplete responses or misunderstandings of the experiment, resulting in 369 valid participants (145 males, average age 19.29 ± 1.51 years). Participants' characteristics were self‐reported. The ASMR screening test was developed with reference to existing studies (Smith et al. 2017; Barratt and Davis 2015). ASMR sensitivity was assessed using the “F” (yes), “S” (not sure), and “J” (no) keys, and the duration of ASMR experience was rated from 1 to 9 (*Never*–*Always*), as detailed in Appendix S1. Trait anxiety was measured using the IAT‐Anxiety paradigm, with the top 15% classified as high anxiety and the bottom 15% as low anxiety. Thirty right‐handed participants were selected. During the experiment, eight participants (seven high anxiety, one low anxiety) found the fNIRS cap uncomfortable, so we streamlined the channels and excluded their data. Two participants withdrew, leaving 30 with near‐infrared data in both anxiety stages. Twenty‐eight participants had data comparing ASMR and non‐ASMR videos, with 14 reporting ASMR experiences during the videos. Descriptive statistics are in Table 1.

**TABLE 1:** Basic information about the participants.

Stage	Group	*N*	Age(/years)	Duration(/s)	Anxiety d‐value	*P*‐value
*n*‐Back	Low‐trait anxiety	19	18.84 ± 1.17	5.74 ± 1.45	−0.85 ± 0.23	< 0.001***
	High‐trait anxiety	19	19.31 ± 1.11	4.32 ± 2.43	0.48 ± 0.23	
State anxiety arousal	Low‐trait anxiety	12	19.1 ± 1.24	5.83 ± 1.59	−0.87 ± 0.24	< 0.001***
	High‐trait anxiety	18	19.33 ± 1.14	4.33 ± 2.50	0.46 ± 0.23	
State anxiety relief	Low‐trait anxiety	10	19.2 ± 1.32	5.80 ± 1.69	−0.85 ± 0.27	< 0.001***
	High‐trait anxiety	18	19.33 ± 1.14	4.33 ± 2.50	0.46 ± 0.23	
ASMR sensation	Low‐trait anxiety	8	18.67 ± 1.32	5.83 ± 1.60	−0.93 ± 0.32	< 0.001***
	High‐trait anxiety	6	19.36 ± 2.36	5.13 ± 2.36	0.46 ± 0.24	

*Note:* “Duration” was the time that participants reported experiencing ASMR when watching ASMR videos; “Anxiety d‐value” is IAT effect, the higher the D‐value, the higher the trait anxiety.

### 2.3 Research Materials and Tools

#### 2.3.1 IAT‐Anxiety Paradigm

We used the IAT‐Anxiety paradigm (Egloff et al. 2005) to gauge trait anxiety. The vocabulary materials included terms related to the self and other categories and words entailing anxiety and calmness (Huang 2012); see Appendix S2 for details.

#### 2.3.2 The Self‐Threatening *n*‐Back Paradigm

We used the self‐threatening *n‐back* paradigm (Balderston et al. 2017) to gradually induce anxiety; the programming materials included self‐threatening sentences “Although most people are right, you are wrong! ! !” in a case where participants completed the *n*‐back task wrongly and simple location pictures.

#### 2.3.3 Videos

We used a whispering ASMR video and a control video (different segments of the same video without the soundtrack) in order to verify the impact of ASMR and time dimensions on anxiety relief; both lasted for 5 min. The video material quoted the whispering video (https://m.youtube.com/watch?v=gs‐iqvX6pOA&t=995s) in the ASMR video library (Liu and Zhou 2019); its stimulus intensity was 3.64 ± 3.32 (on an 8‐point scale). The stimulation duration was 3.64 ± 3.44 min, and the number of persons with effective reports was 14. For more video information, see Appendix S3.

#### 2.3.4 fNIRS

We adopted the NIRSCOUT fNIRS system (NIRx Medizintechnik GmbH, Berlin, Germany), with 16 laser light sources and 16 avalanche diode detectors to collect data. We used a customized data collection interface to gather fNIRS data. The frequency was set at 4.1667 Hz. Appendix S4 depicts the positioning. Covering the frontal lobe and temporal lobe, 45 channels are formed based on all possible adjacent light sources and detectors, and the distance between the light source and the detector is about 2.5 cm. fNIRS recording starts after the breathing training; the resting state recording and analysis time is 2 min; in *n*‐back, the response recording and analysis time is 5 s after each stimulus; ASMR/non‐ASMR video's recording and analysis time is 5 min; the recording and analysis time of the sensation is 4 s before and after the key press, a total of 8 s.

### 2.4 Experimental Procedure

Using the E‐Prime 3.0 demo program, we divided the formal experiment into stages of anxiety arousal and anxiety relief. We adopted the self‐threatening *n*‐back paradigm to elicit the participants' state anxiety. We employed the ABBA design to balance the effect of time on the reduction of state anxiety.

**FIGURE 1:**
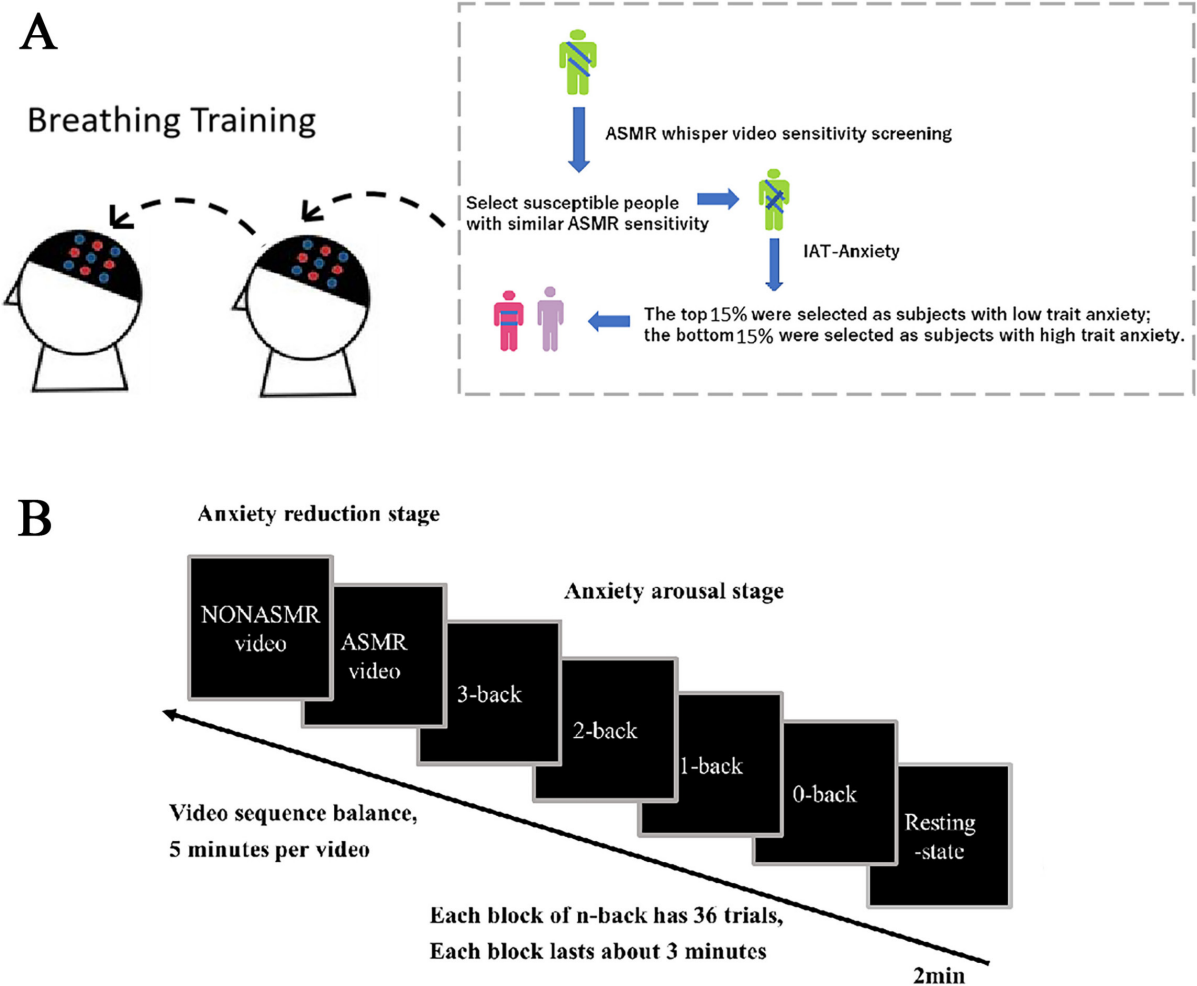
Research process. (A) shows the screening process of participants. (B) is the experimental program diagram.

After the tester fitted the experimental equipment for the participants, the participants were instructed to perform breathing exercises to remain as calm as possible (we did not use any subjective reports of anxiety to avoid giving hints to the participants). Next, the behavioral experiments involving the *n*‐back paradigm (i.e., the anxiety arousal stage) were conducted. Afterward, the participants started watching the video and entered the anxiety relief stage; the order in which participants watched the videos was counterbalanced (i.e., half of the participants watched the ASMR video first, then the non‐ASMR video, and the other half vice versa). Before watching the video, they were asked to respond by pressing the appropriate key when they experienced ASMR sensations such as tingling and numbness. They could press keys several times while watching the video. Figure 1 outlines the research process.

Among them, the *n*‐back task during the anxiety arousal phase consisted of four blocks (0‐back, 1‐back, 2‐back, and 3‐back), each comprising nine trials of a practice experiment followed by 36 trials of a formally presented experiment, with a stimulus presentation time of 5000 ms. Each block began with a detailed instructional message in the practice section, accompanied by both positive and negative feedback regarding the participants' responses. Participants were instructed to respond only to key presses when the specified conditions were met. Upon completing the practice trials, participants were asked to confirm their understanding of the experiment's requirements and could choose to either repeat the practice or proceed to the formal experiment. Once in the formal experiment, participants received feedback solely on errors, which lasted for 2000 ms, while their anxiety arousal was heightened through self‐threatening statements. The entire anxiety arousal phase of the *n*‐back task lasted approximately 20 min. For more details, see Appendix S6.

### 2.5 Data Analysis

#### 2.5.1 Behavioral Data

We used IAT_data_tool_v2.0.0 to analyze the IAT‐Anxiety data. The formula for anxiety d‐value is as follows:

$$d=\frac{\left(\text{mean response time for incompatible tasks}-\text{mean response time for compatible tasks}\right)}{\text{standard deviation of the response time of}\;\mathrm{all}\;\text{trials with correct responses}}$$

The value of d is the IAT effect. The higher the IAT effect, the more anxious the participants' implicit self‐concept (Greenwald et al. 2003); in other words, the trait anxiety will be relatively higher.

#### 2.5.2 fNIRS Data

Using Nirslab, we set event markers for fNIRS signals at 785 and 830 nm and removed motion artifacts from head movement and electrode displacement. We applied band‐pass filtering with 0.2‐ and 0.01‐Hz cutoffs to reduce noise and then used the modified Beer–Lambert law to extract oxy‐Hb and deoxy‐Hb signals. In the Data Viewer module, the plot chart shows the time series of oxy‐Hb (red) and deoxy‐Hb (blue) signals, representing fNIRS data. The map chart illustrates brain activation areas based on hemodynamic data.

In the Data Analysis module, we analyzed segmented fNIRS signals with statistical parametric mapping software. Given oxy‐Hb's greater sensitivity in fNIRS (Zhang et al. 2017), we focused on it for further analysis. We calculated average oxy‐Hb changes in 0‐back and 3‐back tasks, input the data into the General Linear Model to obtain a beta value assuming a 5‐s hemodynamic response function peak (Boynton et al. 2012) and compared condition differences between high‐ and low‐trait anxiety groups using paired sample *t*‐tests. For more details, see Appendix S5.

## 3 Results

### 3.1 Effectiveness in Inducing Anxiety

**FIGURE 2:**
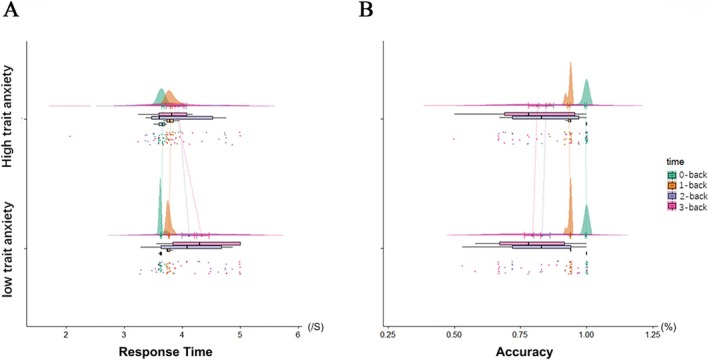
Response time and accuracy in *n*‐back.

In order to verify whether “trait anxiety affects state anxiety” held true for this study, we analyzed the hypothesis through two‐factor repeated measures analysis of variance and corrected using the Greenhouse–Geisser method (the spherical hypothesis was not satisfied). The results showed that task difficulty interacted with high‐ and low‐trait anxiety (ε = 0.58, *F*_(1.74, 0.93)_ = 5.53, *p* = 0.011, *d* = 0.24). A simple effect analysis indicated that during the 3‐back task, the two groups had significant differences in response time (ε = 0.58, *t* = 7.53, *p* = 0.013), but no significant difference in accuracy rate s between the high‐trait anxiety group (0.81 ± 0.15) and the low‐trait anxiety group (0.79 ± 0.13). The accuracy of different task difficulty in the low anxiety group differed significantly (ε = 0.49, *F*_(1.48, 0.34)_ = 25.33, *p* < 0.001). The accuracy of different task difficulty in the high anxiety group also differed significantly (ε = 0.60, *F*_(1.81, 0.22)_ = 17.20, *p* < 0.001). In the low anxiety group, the response time of different task difficulty differed significantly (ε = 0.52, *F*_(1.48, 4.00)_ = 17.76, *p* < 0.001). The accuracy rate and response times of the two groups of participants in blocks of different difficulty levels are displayed in Figure 2; Detailed experimental data are shown in Appendix S7.

### 3.2 Hemodynamic Responses in the Anxiety Arousal Stage

**FIGURE 3:**
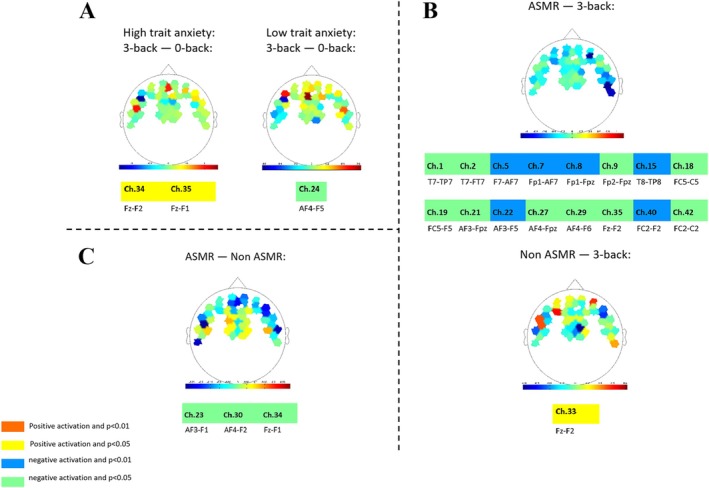
fNIRS results of different stages; (A) shows hemodynamic responses in the anxiety excite stage; (B) shows hemodynamic responses in the anxiety relief stage; (C) shows hemodynamic responses while watching the ASMR video and the non‐ASMR video.

We averaged the fNIRS signals of oxy‐Hb and deoxy‐Hb of the participants on the 0‐back and 3‐back tasks, and we computed the average value of changes of oxy‐Hb in all task periods and the corresponding Beta values. We employed a paired samples *t*‐test to verify that the channel was activated. As seen in Figure 3A, we found that two channels of participants with high trait anxiety were activated, involving the three electrodes Fz, F1, and F2. Channel 24 was activated for participants with low‐trait anxiety (*t* = −2.22, *p* = 0.048) involving the two electrodes of AFz and Fpz. The hemodynamic responses of the dorsolateral prefrontal cortex (Brodmann areas 9 and 10) were significantly reduced.

### 3.3 Hemodynamic Responses in the Anxiety Relief Stage

After the participants completed the *n*‐back task in the anxiety arousal stage, they alleviated their anxiety by watching the ASMR and non‐ASMR videos. The signal we averaged was as presented in Figure 3B; 16 channels were activated, and the areas involving the somatosensory cortex (Brodmann area 5), middle frontal gyrus (part of the prefrontal lobe, Brodmann areas 8 and 46), dorsal prefrontal cortex (Brodmann area 10), temporal lobe (Brodmann area 21), anterior and posterior temporal cross section (Brodmann area 42), and orbitofrontal cortex (Brodmann area 42) were significantly activated.

On the beta test for the participants during the 3‐back task in the post‐anxiety stage, and the participants who watched the non‐ASMR video first, only channel 33 was activated (*t* = 2.16, *p* = 0.047) involving FC6 and C6 electrodes. The hemodynamic responses of the front and back temporal cross sections were significantly enhanced.

### 3.4 Hemodynamic Responses While Watching the ASMR Video and the Non‐ASMR Video Resource Identification Initiative

We did the same with the fNIRS signals when the participants were watching the ASMR and non‐ASMR videos, as depicted in Figure 3C. We found that three channels were activated, involving the five electrodes of AF3, AF4, F1, F2, and Fz. We selected the changes in the oxygenated hemoglobin of the channels activated by watching the ASMR video relative to the non‐ASMR video. When the participants were watching the ASMR video, the hemodynamic responses of the middle frontal gyrus (part of the prefrontal lobe, Brodmann area 8) and dorsolateral prefrontal cortex (Brodmann area 9) were significantly reduced.

### 3.5 Hemodynamic Responses When the Participants Experienced ASMR Sensations Such as Tingling, Numbness

**FIGURE 4:**
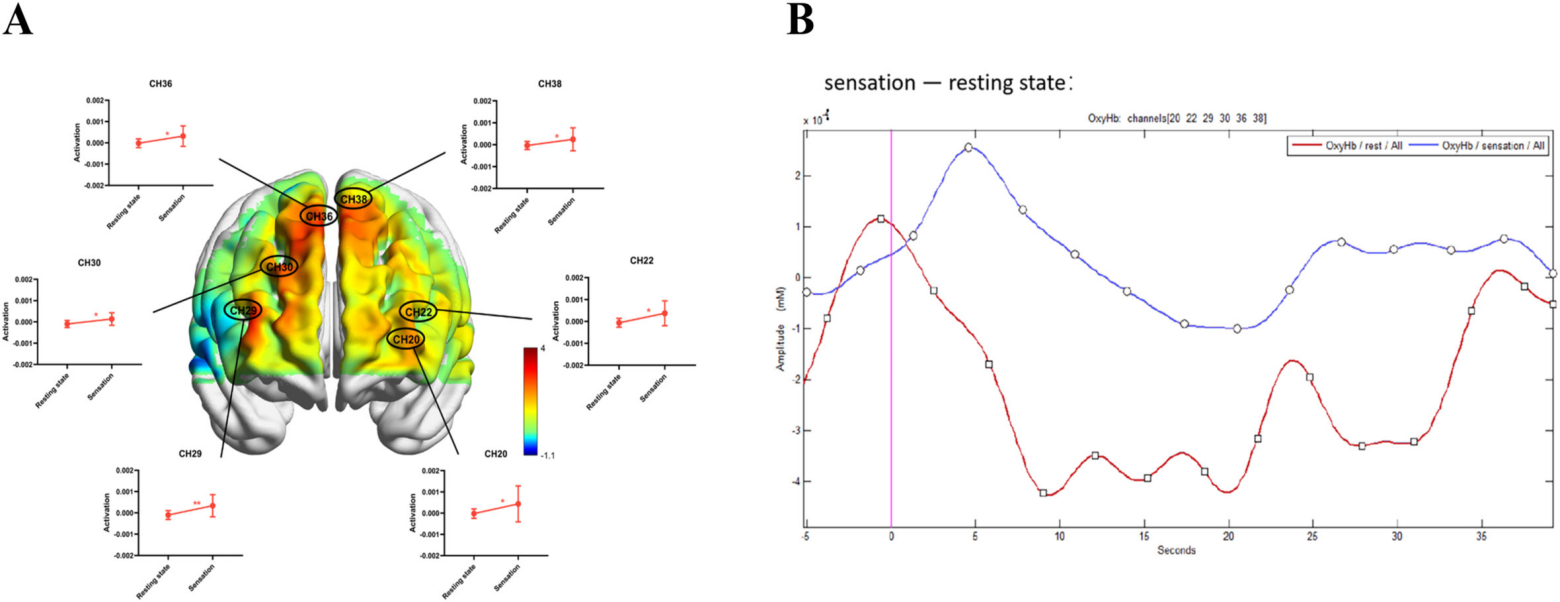
fNIRS results of ASMR sensations. Central: *T*‐test chart of brain activity generated based on paired sample *T*‐test. (a) shows brain activation diagram when experiencing ASMR sensation; (b) is the hemodynamic responses when the participants experienced ASMR sensations. *p < 0.05.

A total of 14 participants reported ASMR sensations such as tingling and numbness by pressing the key while watching the ASMR video. We did the same with the fNIRS signals. We found that six channels were activated, and the hemodynamic responses involving the areas of the frontal lobe (the premotor and auxiliary motor cortices, Brodmann area 6), middle frontal lobe (including the prefrontal area, Brodmann areas 8 and 46), and dorsolateral prefrontal cortex (Brodmann area 9) were significantly reduced. Figure 4A is a brain activation diagram when experiencing ASMR sensation; as shown in Figure 4B, compared with the resting state while watching, oxygenated hemoglobin was increased in the state of reported ASMR.

## 4 Discussion

To explore the effects of ASMR videos on anxiety, we conducted a series of analyses. First, we scrutinized the neural responses of participants with different anxiety levels before and after anxiety arousal in the context of the self‐threatening *n*‐back paradigm, assessing whether the neural patterns elicited during ASMR video viewing (for anxiety reduction) paralleled those observed during anxiety arousal. Second, we compared each individual's neural response while watching the ASMR and non‐ASMR videos. Finally, we sought to delineate the distinctions between brain activity when ASMR “tingling” was induced while watching the ASMR video and the neural response in the resting state.

First of all, in the *n*‐back stage, there was an interaction between high‐ and low‐trait anxiety and task difficulty. As the difficulty of the tasks incremented, the average accuracy rate of the two groups fell significantly, and the average response time rose significantly; this implies that the participants' anxiety was aroused to a certain extent, and that high‐ and low‐trait anxiety affect the degree of arousal of state anxiety (Balderston et al. 2017). Participants with higher trait anxiety tend to underperform on tasks demanding greater cognitive effort (Berggren and Derakshan 2013). However, interestingly, the response time on the 3‐back task of the high trait anxiety group in our study was significantly lower than that of the low‐trait anxiety group, although with no significant difference in accuracy. This is in alignment with the findings of Cooke et al. (2011) and Eysenck et al. (2007), suggesting that anxiety may serve a motivational role, prompting increased effort and potentially enhancing *n*‐back task performance. Another study from Mandrick et al. (2016) found no impact of stress caused by aversive sounds on accuracy‐based *n*‐back performance, suggesting that the effect of stress on accuracy can be enhanced by motivation and cognitive resources. Those with anxiety often allocate excessive attention to and worry about threatening information, which depletes cognitive resources and can impair performance in cognitive activities such as attention control. Studies have pointed out that this may be inaccurate. When task motivation is high, individuals will try to use cognitive resources to normalize performance (Basten et al. 2012). Additionally, the more hurried behavior observed may also be a consequence of heightened anxiety levels. fNIRS monitoring showed that participants with high trait anxiety exhibited a significant upsurge in hemodynamic responses within the middle frontal gyrus, a region of the prefrontal lobe, during the more challenging 3‐back task, while the hemodynamic responses of the dorsolateral prefrontal cortex were obviously depressed in those with low‐trait anxiety. In a study by Tseng et al. (2018), participants engaged in demanding auditory working memory tasks showed a considerable enhancement of hemodynamic responses in the right ventrolateral and preorbital cortices, with activity in the right prefrontal cortex inversely related to the level of state anxiety. Thus, the state anxiety of both the high‐ and low‐trait anxiety participants in our study may be aroused to a certain extent. We propose that the increased activity observed in the middle frontal gyrus under high‐difficulty conditions may be associated with covert speech, such as when participants rehearse the last few items. This activity might also reflect other forms of anxious behavior, including negative self‐talk, particularly among individuals with high trait anxiety, who might be more prone to engage in covert speech during challenging tasks as a strategy to avoid anxiety‐provoking situations (Bramson et al. 2023; Zhang et al. 2024).

After inducing state anxiety, participants who initially watched ASMR videos exhibited significant decreases in hemodynamic responses within key brain areas during the anxiety relief phase. These areas included the somatosensory cortex, the middle frontal gyrus (a part of the prefrontal lobe), the dorsolateral prefrontal cortex, the middle temporal lobe, anterior and posterior temporal regions, and orbital cortex. This finding is consistent with previous studies (Berggren et al. 2013; Duan et al. 2020; Tseng et al. 2018), which indicate that anxiety relief correlates with reduced activation in specific brain regions, particularly those involved in emotional regulation and cognitive processes such as working memory and perceptual load in the frontal lobe. These results support the potential anxiolytic function of ASMR, suggesting that it may modulate neural activity to alleviate anxiety through the engagement of critical networks involved in cognitive and emotional regulation. Nonetheless, it is just as important to consider individual differences and the intricacy of brain networks. Future research should investigate factors such as individual susceptibility to ASMR, cross‐modal interactions, and the long‐term effects of ASMR exposure on brain function to gain a deeper understanding of these dynamics.

Secondly, the hemodynamic responses of participants showed significant differences when watching the ASMR video compared to the non‐ASMR video. Notably, the ASMR video led to a marked decline in hemodynamic responses in the middle frontal gyrus (part of the prefrontal lobe) and the dorsolateral prefrontal cortex. In a separate study, participants who reported experiencing ASMR exhibited reduced resting‐state functional connectivity within the DMN, particularly between frontal, sensory, and attention‐related regions (Smith et al. 2017). Conversely, other studies have suggested that ASMR may actually augment neural activity in the prefrontal cortex. For instance, participants who self‐reported ASMR showed increased activity in the medial prefrontal area, bilateral central anterior gyrus, right upper frontal cortex, left upper temporal cortex, and midline occipital structures while viewing ASMR‐related videos (Smith et al. 2019a, 2019b). Another study identified that ASMR‐induced significant increases in functional connectivity between the posterior cingulate cortex, middle and upper temporal gyrus, cuneiform lobe, and lingual gyrus, as well as enhanced functional connectivity in the medial prefrontal lobe associated with the anterior cingulate cortex (Lee et al. 2020). EEG studies have further demonstrated that ASMR stimulation is associated with increased β1 (sensory motor wave, 12–15 Hz) band activity in the frontal region, γ1 in the central area, and γ2 in the frontoparietal areas of both hemispheres (Seifzadeh et al. 2021). In our study, both the ASMR and non‐ASMR videos were presented after inducing anxiety. This suggests that ASMR's impact on cerebral blood oxygenation is influenced by anxiety, with ASMR more effectively alleviating prefrontal neural activity associated with anxiety compared to the non‐ASMR video. The ecological validity of our experimental design is crucial for these findings, as ASMR is highly subjective and commonly experienced in real‐world contexts, making it challenging to replicate in controlled laboratory settings (Mahady et al. 2023). To address this, we utilized fNIRS to measure brain activity in a manner that closely mirrors real‐world ASMR experiences, thereby providing a more accurate representation of its neural mechanisms (Chen et al. 2020).

The fNIRS data analysis from our study disclosed that during ASMR experiences, the hemodynamic responses in the frontal lobe—specifically in the premotor and supplementary motor cortices, the middle frontal gyrus, and the dorsolateral prefrontal cortex—were significantly reduced compared to baseline resting states. These results align with the fMRI study conducted by Lochte et al. (2018), which showed heightened activity in the medial prefrontal cortex during ASMR video viewing and tingling sensations. The diminished hemodynamic responses in the prefrontal regions may indicate a state of relaxation or decreased cognitive load, though these changes may also be linked to various cognitive or emotional processes occurring alongside ASMR. Importantly, the prefrontal cortex is integral to the regulation of attention, emotional processing, and self‐awareness, indicating that the observed changes may reflect a reconfiguration of these functions. These outcomes are concordant with previous EEG and fMRI studies examining ASMR, especially in the context of state anxiety and prefrontal cortex involvement. Nevertheless, it is important to consider alternative interpretations. Given the multifaceted nature of the prefrontal cortex and its diverse functions, the detected changes might be influenced by individual differences in cognitive strategies, subjective interpretations of the video content, or participants' baseline anxiety levels.

A significant observation from this study pertains to the middle frontal gyrus, which exhibited heightened hemodynamic activity during the anxiety arousal phase. However, there was a marked decrease in hemodynamic activity during the anxiety remission phase while participants reported experiencing ASMR. Although prior research (e.g., Zhang et al. 2022; Pfurtscheller et al. 2021) suggests that increased activity in this region may correlate with elevated anxiety levels, given the critical role of the middle frontal gyrus in cognitive control, working memory, and the integration of sensory information, we suggest that changes in this activity may not only be associated with fluctuations in anxiety levels but may also reflect dynamic changes in cognitive and affective states during the ASMR experience. In particular, reductions in frontal middle gyrus activity during ASMR may be associated with a redistribution of attention, an enhanced state of relaxation, or a deeper immersion in sensory stimuli, and these processes may work together to promote anxiety relief.

The present study provides empirical support for the hypothesis that ASMR videos can alleviate anxiety, as evidenced by both behavioral and neurophysiological data. Using the self‐threatening *n*‐back task alongside ASMR and non‐ASMR video stimuli, we observed significant changes in hemodynamic responses measured via fNIRS, particularly during heightened state anxiety. The self‐threatening *n*‐back task successfully elicited anxiety across participants with varying trait anxiety levels. Our findings suggest that ASMR videos play a role in reducing anxiety. Additionally, the results point to the calming effects of ASMR that appear to be correlated with the modulation of brain regions associated with anxiety and cognitive load, particularly in the prefrontal cortex. This contributes to the literature supporting ASMR as a potential intervention for anxiety.

### 4.1 Limitations and Future Directions

Limitations of the study should be addressed. First, in the current investigation, ASMR sensitivity was assessed solely through self‐reported measures by participants. However, considering the specific brain regions activated by ASMR, as evidenced by fNIRS, it is imperative that future research endeavors to correlate these regions with relevant cognitive functions. This could involve the development of standardized questionnaires or the identification of other comprehensive and precise assessment tools to evaluate ASMR sensitivity among participants. *Second, the lack of any auditory stimuli in the current control condition could confound comparisons between the ASMR and control conditions by non‐ASMR auditory responses in the ASMR condition. Future research should address this limitation by incorporating a more consistent control condition*.

Furthermore, subsequent studies should aim to differentiate between neural responses associated with state anxiety and those linked to cognitive processes, such as working memory and executive function. We intend to employ more advanced experimental designs and data analysis methodologies, including a combination of fMRI and EEG, to elucidate these phenomena. This multimodal approach will facilitate a more precise identification of the neural correlates of state anxiety and cognitive processes, thereby deepening our insight into the neural pathways by which ASMR affects anxiety and cognitive function. In summary, our findings provide preliminary evidence supporting the efficacy of ASMR in mitigating anxiety. Future research should explore the potential influence of personality traits on the effectiveness of ASMR in anxiety reduction, as well as the impact of specific ASMR content on this effectiveness. Such investigations could assist individuals with varying personality traits in selecting appropriate ASMR videos tailored to their needs.

## 5 Conclusions

In the anxiety relief stage, while watching the ASMR video, the participants' brain responses (the hemodynamic responses of the middle frontal gyrus and dorsolateral prefrontal cortex were significantly reduced) were opposite to those when anxiety was aroused in participants with high trait anxiety (the hemodynamic responses of the middle frontal gyrus, part of the prefrontal lobe, were significantly enhanced).

The participants' reported experiences of ASMR while watching the ASMR video once again prove the unique neural activation related to ASMR. For the first time, we have connected ASMR‐related activation with anxiety‐related activation, which provides certain neurophysiological evidence for using ASMR videos to relieve anxiety.

## Author Contributions

**Huan Jiang:** conceptualization, formal analysis, investigation, writing – original draft. **Yating Chen:** data curation, supervision, validation, writing – review and editing. **Feng Guo:** data curation, methodology, supervision. **Binjie Yang:** data curation, software, writing – review and editing. **Jiani Yan:** formal analysis, writing – original draft. **HuiLing Li:** project administration, writing – review and editing. **Ke Jiang:** conceptualization, supervision. **Qiang Zhou:** conceptualization, methodology. **Xianghe Zhu:** conceptualization, resources, writing – review and editing.

## Ethics Statement

Our study was approved by an ethics committee, the ethical lot number is 2020‐006, and the approval unit is the Ethics Committee of Wenzhou Medical University.

## Conflicts of Interest

The authors declare no conflicts of interest.

## Peer Review

The peer review history for this article is available at https://www.webofscience.com/api/gateway/wos/peer‐review/10.1111/ejn.70251.

## Supporting information


**Appendix S1:** Supporting information.

**Appendix S2:** Supporting information.

**Appendix S3:** Supporting information.

**Appendix S4:** Supporting information.

**Appendix S5:** Supporting information.

**Appendix S6:** Supporting information.

**Appendix S7:** Supporting information.

## Data Availability

The data that support the findings of this study are openly available in data for ASMR.zip (https://www.researchgate.net/publication/360256822_data_for_ASMR).
