# Supplementary material for: An Exploratory Study on the Regulatory Effect of Autonomous Sensory Meridian Response on Anxiety: Evidence From Functional Near‐Infrared Brain Imaging Technology
Source: Eur J Neurosci. 2025 Sep 14;62(5):e70251. doi: 10.1111/ejn.70251 (PMC12434388; doi:10.1111/ejn.70251)
Supplement: Supplementary file 1 — Appendix S1: Supporting information. [file EJN-62-0-s006.pdf]

395 college students' self-reported response to the ASMR videos

Video 1—ASMR-(0-no,1-unsure,2-yes)											
sub	ASMR	duration	nervous	like	relax	calm	pay attention	sex impulse	comfortable	safe	anxious
1	0	1	4	4	2	2	6	4	2	4	4
2	1	2	4	4	3	3	5	3	3	3	3
3	1	6	3	5	5	5	6	4	4	5	2
4	1	3	4	4	4	3	4	4	4	4	4
5	1	7	6	2	3	3	5	4	2	4	4
6	2	1	1	5	5	5	5	3	3	4	4
7	2	3	1	4	4	4	6	1	4	4	4
8	0	1	4	4	4	4	4	4	4	4	4
9	1	3	4	4	4	4	5	4	4	4	4
10	2	5	2	5	5	5	3	4	3	3	3
11	0	2	5	3	4	3	4	6	5	3	5
12	0	4	6	1	1	2	5	1	1	1	5
13	2	3	2	5	2	4	4	4	2	4	4
14	0	2	4	4	5	5	4	4	4	5	4
15	0	2	4	4	3	4	3	4	4	3	4
16	0	1	3	4	5	4	5	4	4	4	4
17	1	2	3	4	5	4	5	4	5	5	2
18	2	6	3	4	5	2	6	4	4	4	5
19	1	5	5	4	3	3	5	4	4	4	5
20	1	3	3	3	3	3	3	3	3	3	3
21	1	9	5	3	3	3	4	4	2	3	5
22	2	4	3	5	5	4	5	4	3	3	4
23	0	2	3	4	4	3	3	3	3	3	3
24	1	1	3	4	3	3	4	4	3	3	4
25	2	6	6	3	3	2	4	5	2	4	5
26	2	3	5	4	4	4	5	4	4	4	5
27	1	2	2	5	5	6	5	4	4	5	3
28	2	3	3	4	5	6	5	4	5	4	3
29	1	6	5	4	3	3	4	4	3	4	4
30	0	2	5	5	3	3	2	4	2	2	5
31	1	5	1	4	7	7	6	4	7	7	1
32	0	2	1	4	4	4	6	4	4	4	4
33	2	4	2	6	6	6	6	4	6	6	2
34	1	5	4	5	2	3	6	1	2	3	3
35	2	2	5	4	6	5	4	4	3	4	4
36	2	8	5	4	3	3	6	4	3	3	5
37	0	5	5	5	5	5	6	6	5	5	5
38	0	1	3	3	3	3	3	3	3	3	3
39	0	4	5	3	4	3	5	5	5	5	4
40	1	3	4	4	4	3	2	4	4	3	3
41	1	3	4	4	5	3	4	4	5	4	4
42	0	2	5	4	5	5	4	4	4	4	4
43	0	1	1	4	4	4	5	4	3	4	4
44	0	2	4	4	4	4	4	3	4	4	5
45	1	2	5	5	5	3	3	5	5	4	3
46	2	3	4	5	5	5	3	4	5	5	4
47	2	3	2	6	6	6	2	2	7	6	1
48	1	1	3	3	5	6	2	4	6	4	3
49	2	5	4	6	7	6	5	4	5	4	3
50	0	2	4	4	4	4	5	5	4	4	4
51	1	7	6	2	3	5	2	6	6	5	5
52	1	5	5	2	2	3	5	5	2	3	4
53	2	3	3	3	3	3	3	3	4	3	3
54	1	3	2	4	5	5	3	4	4	4	3
55	2	6	5	1	1	3	1	3	1	1	6

56	2	3	2	6	6	6	7	4	6	7	2
57	1	3	4	4	4	3	3	3	3	4	3
58	2	6	5	4	5	5	4	4	5	5	5
59	2	7	5	3	2	5	5	5	3	2	6
60	1	8	5	5	4	5	5	4	5	4	3
61	1	5	4	5	6	6	5	4	5	4	4
62	2	4	1	4	5	6	3	4	5	4	2
63	1	4	4	4	3	5	6	1	3	3	4
64	0	1	4	5	2	3	4	4	3	3	3
65	2	4	5	3	3	4	4	4	3	4	4
66	0	2	4	2	4	4	5	4	2	3	5
67	2	6	5	4	3	5	3	2	3	4	5
68	2	3	3	3	3	3	4	1	3	2	4
69	0	1	3	3	2	3	6	3	3	3	3
70	2	5	2	4	5	5	6	4	6	6	2
71	2	4	5	3	4	5	6	1	4	5	5
72	2	3	3	3	3	4	4	3	3	3	4
73	2	6	5	3	4	6	5	4	4	4	2
74	2	2	2	3	5	5	6	4	4	5	2
75	1	1	2	2	5	4	5	1	4	4	4
76	1	3	1	4	1	4	2	4	3	3	4
77	1	1	1	6	6	7	5	5	6	6	1
78	0	1	4	4	4	4	4	1	4	4	4
79	0	1	4	4	4	4	4	4	4	4	4
80	0	1	1	1	5	5	6	4	4	4	4
81	0	2	3	3	3	3	5	4	5	4	2
82	0	1	4	4	5	5	3	4	6	5	3
83	0	1	3	3	2	5	6	3	3	3	3
84	2	9	2	5	1	2	6	3	6	5	2
85	2	4	2	6	6	5	6	4	7	5	2
86	0	2	5	3	3	3	5	4	2	4	4
87											
88	1	1	4	4	4	6	6	3	3	3	4
89	2	3	4	4	3	3	4	3	4	4	5
90	2	5	5	5	5	2	2	3	3	3	5
91	1	1	3	3	2	3	6	5	5	4	5
92	2	4	3	4	6	2	3	4	5	4	4
93	2	1	3	3	3	5	6	3	3	3	3
94	0	1	2	4	4	4	4	4	4	4	4
95	1	2	4	4	2	3	6	5	4	3	4
96	1	7	3	3	3	3	3	3	3	3	3
97	2	7	5	4	5	4	5	4	5	4	3
98	0	1	1	3	5	5	6	4	3	4	3
99	1	5	5	4	4	5	6	4	4	5	4
100	0	1	4	4	4	4	4	4	4	4	4
101	0	1	4	5	4	5	5	4	4	4	3
103	1	2	4	4	5	5	3	4	5	5	4
104	1	6	6	2	3	2	4	5	2	4	5
105	1	6	6	1	1	2	3	4	2	2	2
106	2	2	5	4	3	3	2	6	2	1	2
107	2	5	2	1	1	1	1	1	5	4	5
108	2	7	2	5	5	4	6	5	5	2	4
109	0	3	3	3	3	3	3	3	3	3	3
110	1	4	3	4	5	3	5	4	4	3	3
111	2	6	2	5	6	6	7	4	6	4	2
112	0	1	3	4	3	4	3	4	4	3	4
113	0	5	4	4	5	5	3	4	6	4	4
114	1	5	3	6	5	2	6	5	3	3	5

115	0	1	4	4	4	4	5	4	5	4	4
116	0	2	3	4	5	7	7	6	5	3	3
117	1	3	4	4	4	4	5	5	5	4	4
118	1	9	6	5	3	3	5	5	5	4	4
119	0	2	3	3	5	5	3	3	3	3	3
120	1	4	5	3	1	2	4	4	1	1	2
121	2	2	1	2	2	2	4	2	4	4	4
122	2	3	3	6	5	6	3	4	5	2	6
123	0	2	3	5	5	4	6	4	4	4	4
124	2	3	3	6	5	6	4	4	6	5	4
125	1	3	3	3	4	5	6	4	2	4	5
126	1	4	5	5	6	7	6	4	5	5	3
127	1	1	4	4	5	5	5	5	5	5	4
128	2	6	4	4	4	4	5	3	3	3	3
129	0	1	4	4	4	4	3	4	4	4	4
130	2	7	4	2	4	4	4	4	2	4	5
131	2	1	4	3	4	4	3	4	3	4	4
132	1	7	5	4	3	4	5	4	3	4	4
133	0	4	1	2	4	4	5	4	4	4	4
134	1	4	5	2	3	5	3	5	3	3	5
135	1	6	3	3	5	4	3	3	5	4	2
136	2	4	4	5	5	5	5	1	5	5	4
137	2	4	5	4	3	6	6	4	4	4	4
138	2	4	4	4	4	4	4	4	4	4	4
139	1	3	5	4	5	4	5	4	5	3	4
140	2	7	6	1	2	2	5	4	1	3	6
141	2	8	2	6	6	5	4	4	6	4	3
142	2	6	1	4	4	5	3	4	4	4	5
143	0	1	4	5	3	4	4	6	4	5	4
144	2	7	4	4	3	3	3	4	3	4	5
145	0	4	4	4	4	4	5	4	5	5	4
146	1	1	4	5	5	5	4	5	5	4	4
147	0	2	4	5	5	4	1	2	4	5	5
148	2	1	4	4	4	4	3	4	2	2	4
149	1	1	4	4	5	6	5	4	4	4	4
150	2	6	1	4	4	4	4	4	4	4	1
151	0	1	4	4	6	6	5	4	6	4	2
152	0	2	4	5	6	5	4	4	6	6	3
153	1	5	5	3	5	3	6	1	1	2	6
154	0	2	5	5	2	2	6	5	2	2	4
155	0	1	4	4	4	4	4	5	5	5	4
156	2	2	1	6	6	6	3	3	6	5	4
157	1	1	2	5	6	6	5	1	6	4	1
158	0	2	4	4	3	2	6	4	5	4	2
159	1	1	3	4	4	4	4	4	4	4	4
160	2	5	1	5	6	6	4	4	7	5	2
161	0	2	3	3	5	6	4	4	5	5	2
162	0	4	2	3	5	3	6	5	4	5	4
163	0	1	4	4	4	5	5	4	4	4	4
164	2	2	4	3	5	5	5	2	3	3	2
165	0	5	5	4	4	4	4	4	4	4	4
166	2	6	2	5	3	3	5	4	5	5	3
167	1	1	4	5	5	2	5	5	5	5	3
168	0	3	3	5	5	2	3	4	4	4	3
169	0	2	4	6	6	6	6	5	7	6	4
170	0	1	4	4	4	4	6	4	5	4	4
171	0	1	1	5	5	3	4	6	6	6	1
172	0	1	3	4	4	3	4	4	4	4	4

173	0	4	4	4	3	3	5	1	4	4	3
174	0	5	1	4	1	1	1	1	4	1	1
175	0	1	4	4	4	4	4	4	4	4	4
176	1	1	4	4	4	5	5	4	4	4	4
177	0	1	3	3	3	4	4	3	4	4	4
178	0	1	2	3	2	3	3	3	3	4	4
179	0	2	3	3	2	3	3	3	5	3	3
180	0	3	3	3	3	3	3	3	4	4	4
181	1	1	1	7	7	4	6	4	6	4	2
182	0	1	3	4	5	5	5	4	4	4	4
183	0	1	3	3	3	3	5	4	3	3	3
184	0	1	5	4	3	3	4	4	4	4	5
185	2	3	2	3	4	4	5	4	2	4	4
186	1	5	1	5	6	6	5	4	7	4	4
187	1	1	4	4	4	4	4	4	4	4	4
188	1	2	3	3	6	2	4	3	6	6	4
189	1	1	3	3	3	2	3	3	2	2	3
190	2	6	4	3	4	5	6	5	4	4	3
191	2	1	2	5	5	5	4	4	6	5	3
192	2	4	4	4	4	4	4	4	5	4	4
193	1	8	6	3	2	2	5	5	3	5	6
194	2	5	4	5	5	5	4	4	5	5	3
195	1	7	5	4	4	5	5	5	4	3	4
196	0	1	3	3	4	4	5	3	3	3	3
197	1	2	3	4	4	4	4	3	4	1	1
198	0	2	4	4	4	3	4	4	4	4	4
199	2	6	4	4	4	4	4	4	4	4	4
200	1	3	3	4	3	3	4	3	3	4	4
201	2	5	2	5	6	6	6	5	6	5	3
202	2	2	1	7	7	4	5	5	7	5	3
203	2	1	5	3	4	5	4	4	5	4	4
204	2	6	5	5	3	3	5	5	3	3	5
205	2	3	4	2	3	2	6	4	3	4	5
206	1	8	3	5	6	5	4	5	6	5	2
207	2	6	6	6	1	1	6	4	1	1	5
208	2	6	5	2	3	3	5	4	3	3	5
209	1	2	4	4	5	4	5	4	4	3	4
210	1	2	4	3	4	4	4	4	3	4	4
211	0	1	4	4	4	4	4	4	4	4	4
212	2	6	5	4	2	2	4	4	3	4	4
213	1	3	4	3	4	3	3	3	4	3	5
214	1	2	4	4	5	4	5	4	4	4	4
215	0	3	2	6	4	4	2	4	5	5	2
216	0	1	4	4	4	4	5	4	4	4	4
217	2	3	4	4	4	4	4	4	4	4	4
218	0	2	2	4	4	4	4	4	4	4	4
219	1	5	6	1	2	2	5	4	2	4	4
220	0	1	4	4	5	4	4	4	5	4	4
221	0	1	4	4	4	5	5	6	4	4	4
222	0	1	4	4	4	4	4	4	4	4	4
223	0	1	1	4	4	4	4	4	4	4	4
224	0	1	1	4	4	4	4	4	4	4	4
225	1	2	4	4	4	6	5	4	4	4	4
226	0	1	4	4	4	4	4	4	4	4	4
227	1	1	4	4	4	4	5	4	4	4	4
228	0	4	4	4	4	4	5	4	3	4	4
229	0	1	4	4	4	4	4	4	4	4	4
230	0	5	4	4	4	4	6	4	4	4	4

231	0	1	4	4	4	4	4	4	4	4	4
232	0	1									
233	0	1	4	4	4	4	4	4	4	4	4
234	1	3	4	4	4	4	4	4	4	4	4
235	1	3	4	4	4	4	4	4	4	4	4
236	2	1	4	4	4	4	5	4	4	4	3
237	0	1	4	4	4	4	4	4	4	4	4
238	0	1	3	3	3	3	3	3	3	3	3
239	0	1	2	2	2	2	2	3	2	4	4
240	0	1	1	4	4	4	4	5	5	4	4
241	0	2	4	4	4	4	4	4	5	4	4
242	0	1	3	3	3	3	4	3	3	3	3
243	1	1	4	4	4	4	4	4	4	4	4
244	2	1	4	4	5	3	5	6	4	2	4
245	2	1	1	1	1	1	4	4	4	4	4
246	2	5	5	5	5	5	4	3	5	5	3
247	1	4	3	3	3	3	3	3	4	3	3
248	2	7	1	6	7	6	6	6	6	6	2
249	0	2	2	4	4	4	5	4	4	4	4
250	0	2	2	5	6	6	6	5	6	6	2
251	0	2	3	4	4	5	6	4	5	4	4
252	0	1	1	4	4	5	5	5	4	4	4
253	1	1	1	4	5	5	5	4	4	5	4
254	0	2	3	3	4	5	6	2	3	4	2
255	0	3	4	4	5	6	5	4	3	4	3
256	0	2	3	4	5	4	5	4	5	4	4
257	1	2	1	2	4	4	4	4	4	4	4
258	1	4	5	4	3	5	5	4	5	5	3
259	2	6	4	6	4	4	4	4	5	4	4
260	2	3	4	4	4	4	5	5	5	4	4
261	2	9	6	5	4	5	4	4	5	4	4
262	2	1	3	3	3	4	3	3	3	3	4
263	2	1	3	4	3	4	5	3	4	2	4
264	2	1	3	5	5	5	4	4	5	4	4
265	2	9	6	5	3	4	3	2	4	5	4
266	2	9	6	4	4	4	5	4	3	4	4
267	2	9	6	5	5	4	5	4	4	4	5
268	1	3	4	4	5	4	4	4	5	4	3
269	2	8	3	5	6	4	5	5	6	4	3
270	1	2	4	5	5	3	4	2	5	4	4
271	2	8	3	3	5	3	5	3	4	5	2
272	1	7	6	4	3	3	6	3	4	5	3
273	1	4	2	4	3	2	4	4	5	4	5
274	2	3	4	3	5	6	6	3	4	3	2
275	0	5	4	4	4	4	4	4	4	4	4
276	1	1	3	1	1	1	1	4	3	3	5
277	0	1	4	4	4	4	4	4	4	4	4
278	1	7	6	3	3	4	5	4	3	4	4
279	2	4	5	4	3	5	5	3	4	3	4
280	1	4	6	2	3	3	5	4	3	4	5
281	2	2	2	4	3	3	5	4	3	3	4
282	0	2	4	2	5	5	4	4	2	3	5
283	0	3	3	4	3	4	4	4	4	4	4
284	0	1	3	3	3	4	3	3	3	3	3
285	0	2	3	3	4	5	5	3	3	4	3
286	1	1	4	4	4	4	4	4	4	4	4
287	0	1	4	4	4	5	4	4	4	4	4
288	1	3	4	4	4	3	4	4	4	4	4

289	2	2	4	4	4	4	5	4	4	4	4
290	0	1	4	4	4	4	4	4	4	4	4
291	2	1	4	4	4	4	5	4	4	4	4
292	2	7	4	5	5	5	5	5	4	5	4
293	2	3	1	4	5	5	3	4	6	5	4
294	1	1	1	4	5	4	4	1	4	4	3
295	0	1	4	4	4	4	4	4	4	4	4
296	2	8	5	1	1	1	4	1	1	4	7
297	2	5	3	4	5	5	4	4	4	4	4
298	1	6	1	5	1	2	2	7	6	4	1
299	0	2	4	4	4	4	4	3	4	4	5
300	0	1	3	3	3	3	3	3	3	3	3
301	2	3	2	5	6	6	6	4	6	5	2
302	0	2	1	4	4	4	5	4	4	4	4
303	0	3	3	3	2	3	3	3	2	3	5
304	1	2	4	4	4	4	2	4	4	4	4
305	2	2	4	4	4	4	4	4	4	4	4
306	1	1	3	4	3	5	5	4	5	4	3
307	2	7	2	5	5	5	5	4	5	5	3
308	0	1	4	4	4	4	5	4	4	4	4
309	0	1	4	4	4	4	5	4	4	4	4
310	1	3	4	4	3	4	5	4	4	3	3
311	2	7	2	4	4	5	6	4	5	4	2
312	1	1	5	4	2	2	6	4	2	2	6
313	0	2	3	3	4	4	4	4	5	5	3
314	1	1	4	4	4	4	4	4	4	4	4
315	2	5	5	4	2	3	6	5	3	3	4
316	1	7	6	7	3	4	6	6	4	4	3
317	0	1	5	4	4	3	4	4	4	4	5
318	2	5	3	5	6	5	4	4	5	4	4
319	2	1	1	1	7	4	7	5	5	4	3
320	2	2	3	4	4	4	4	4	4	4	4
321	0	1	4	4	4	4	2	4	3	4	4
322	2	2	1	1	4	4	4	4	4	4	4
323	1	1	1	7	6	6	7	2	6	7	1
324	1	1	3	4	4	4	5	4	4	4	4
325	1	1	4	5	6	5	6	6	5	5	2
326	1	3	3	5	5	5	6	2	5	4	3
327	2	3	4	5	4	4	4	5	5	4	4
328	1	2	3	3	3	3	3	3	3	3	3
329	1	2	5	4	3	3	3	6	3	4	5
330	1	1	4	4	4	4	4	4	6	4	4
331	0	6	5	5	3	3	5	2	3	3	4
332	1	1	6	6	6	6	7	6	3	5	5
333	2	5	6	5	1	3	5	4	3	4	6
334	1	1	2	5	5	5	3	4	5	5	2
335	0	4	5	2	3	3	4	2	2	2	3
336	2	1	4	7	4	5	6	1	7	5	2
337	2	8	4	3	3	3	4	5	3	3	4
338	1	1	4	4	5	5	3	4	4	4	3
339	0	1	5	4	4	4	4	4	4	4	4
340	0	3	5	4	3	5	6	4	5	3	3
341	0	1	1	3	6	6	3	3	3	3	3
342	0	2	5	3	2	2	3	3	2	3	4
343	2	2	5	2	3	5	5	2	3	3	5
344	2	4	7	1	1	1	3	4	1	1	7
345	1	7	5	1	2	2	3	3	3	3	4
346	1	4	4	4	3	3	5	4	3	3	4

347	2	2	3	3	1	2	4	4	2	3	3
348	1	3	5	3	4	5	6	4	4	4	3
349	2	6	6	5	5	5	3	5	5	2	3
350	0	4	6	6	6	3	5	4	6	4	3
351	0	2	1	4	2	6	4	1	4	2	3
352	2	4	4	5	5	5	5	6	4	4	4
353	2	9	3	1	1	4	2	4	2	3	5
354	1	1	4	4	4	6	5	4	3	5	4
355	2	7	2	6	5	4	4	4	6	4	3
356	2	2	1	4	5	4	4	4	1	4	4
357	2	8	4	4	4	4	5	4	3	4	4
358	1	2	4	4	4	5	5	4	3	4	6
359	0	2	5	5	4	6	5	5	5	4	3
360	1	7	6	2	2	4	6	1	2	4	3
361	2	2	4	4	4	4	4	4	5	4	4
362	2	1	5	4	5	5	5	4	4	4	4
363	1	3	2	4	6	7	6	4	5	4	4
364	0	1	3	4	3	3	4	1	2	2	3
365	0	2	4	4	5	6	6	3	5	6	2
366	2	6	3	4	4	4	4	4	3	3	3
367	1	2	4	4	4	4	4	4	4	4	4
368	2	4	2	3	2	6	7	6	7	6	2
369	2	2	4	5	6	5	3	4	5	4	4
370	1	4	3	3	3	3	3	3	3	3	3
371	1	2	5	4	3	4	5	3	3	3	4
372	1	3	4	4	4	5	3	4	6	5	3
373	2	4	6	3	5	5	5	5	4	5	3
374	1	1	3	2	5	4	5	4	3	4	4
375	2	3	4	4	4	3	4	4	3	4	4
376	0	2	5	1	1	1	4	4	1	1	7
377	2	3	3	3	5	5	2	3	5	3	2
378	1	2	4	4	5	5	5	4	5	4	3
379	2	3	4	4	5	5	4	4	5	4	3
380	2	8	4	4	5	5	4	4	5	4	3
381	2	2	4	4	3	5	4	4	2	4	4
382	0	3	4	2	2	3	2	4	3	2	3
383	0	1	4	4	4	3	4	4	3	4	4
384	0	2	4	2	4	3	2	4	3	4	5
385	2	3	3	3	4	5	3	3	3	3	5
386	2	5	3	4	4	5	4	4	5	4	4
387	2	7	5	7	7	5	5	6	7	7	1
388	2	1	4	4	4	3	4	4	4	4	3
389	0	4	6	1	4	5	6	5	1	1	5
390	2	8	5	4	4	4	4	4	3	4	5
391	0	5	4	2	3	3	4	4	3	4	4
392	2	7	1	4	4	4	4	4	2	3	3
393	0	2	5	3	3	4	3	4	3	3	4
394	2	1	4	4	4	4	5	4	4	4	4
395	1	3	4	4	3	3	2	4	2	3	5

Video 2—ASMR-(0-no,1-unsure,2-yes)											
sub	ASMR	duration	nervous	like	relax	calm	pay attention	sex impulse	comfortable	safe	anxious
1	1	1	5	2	2	2	2	4	2	4	5
2	2	2	3	3	4	4	3	3	5	5	3
3	1	8	6	4	5	5	6	4	5	3	3
4	1	4	4	4	3	4	5	4	3	4	4
5	1	4	5	3	5	3	5	4	3	4	4
6	2	1	4	4	5	5	5	4	4	4	2
7	2	2	4	4	4	4	4	4	5	4	4
8	0	1	4	4	4	4	4	4	4	4	4
9	1	3	4	4	4	5	5	4	4	4	4
10	2	4	3	3	3	4	5	4	5	5	5
11	1	3	6	5	3	2	3	6	5	3	6
12	0	7	7	1	1	2	2	1	1	1	6
13	2	7	3	6	5	6	2	4	5	4	4
14	0	1	4	3	3	3	3	4	3	3	4
15	0	1	5	4	4	4	5	4	3	4	4
16	0	1	3	3	4	4	4	3	4	4	4
17	1	2	3	5	6	5	5	4	5	6	3
18	2	4	5	4	3	3	6	4	3	4	5
19	1	5	5	4	3	3	4	4	4	3	5
20	2	4	3	3	4	3	4	4	4	3	4
21	2	9	3	2	2	2	4	4	2	2	5
22	2	5	5	3	3	3	5	4	3	3	5
23	0	1	3	3	5	3	3	3	3	3	3
24	2	3	3	4	4	3	4	4	3	3	4
25	2	8	6	3	2	2	5	5	4	3	5
26	0	1	4	3	3	4	5	4	4	4	4
27	0	2	4	4	3	5	5	4	4	4	4
28	2	5	4	4	5	5	5	4	5	4	4
29	1	2	4	4	4	4	4	4	4	4	3
30	2	6	6	2	2	2	1	4	2	2	6
31	1	6	6	2	2	2	2	2	2	3	5
32	1	7	4	4	3	3	3	4	2	3	5
33	2	8	3	6	6	7	6	4	6	6	2
34	0	1	2	3	4	7	4	4	4	6	2
35	0	1	3	3	5	5	4	3	4	4	4
36	2	8	5	3	3	3	4	4	3	3	5
37	1	5	6	5	5	5	5	5	5	5	5
38	0	1	3	3	3	3	3	3	3	3	3
39	1	7	3	3	3	5	3	6	5	4	2
40	2	4	3	4	4	5	5	4	5	5	5
41	1	4	4	5	4	3	4	4	5	4	3
42	0	2	4	4	5	4	4	4	3	4	4
43	0	1	3	3	4	5	5	4	5	4	4
44	0	4	4	4	5	5	5	3	4	5	4
45	1	2	2	5	5	3	3	4	6	4	2
46	2	4	4	5	5	5	3	4	5	5	3
47	2	2	2	6	6	6	3	3	6	6	2
48	1	3	3	5	5	5	3	4	6	5	3
49	2	6	4	4	3	3	2	1	3	3	4
50	0	2	4	4	5	4	5	5	4	4	4
51	1	7	5	5	5	5	5	5	6	6	5
52	1	5	5	3	2	3	3	4	3	3	5
53	2	7	5	5	2	3	4	4	5	4	4
54	1	3	3	4	5	5	5	4	5	4	3
55	2	5	5	1	1	1	1	1	1	1	7

56	2	5	1	7	7	7	6	4	6	7	3
57	2	2	2	5	5	5	5	3	5	4	3
58	0	1	2	4	2	2	3	3	5	5	2
59	2	8	6	2	6	3	2	6	2	2	5
60	1	6	4	3	4	3	4	3	4	5	4
61	0	1	4	4	3	7	5	4	2	4	4
62	2	4	4	3	4	5	3	4	5	6	3
63	1	2	3	2	5	4	4	1	6	6	3
64	0	1	3	3	2	2	2	4	3	3	3
65	2	4	3	3	3	3	3	4	3	4	4
66	0	2	5	1	2	3	2	4	2	3	5
67	2	4	2	4	4	5	5	2	4	4	3
68	2	3	2	2	2	4	3	1	1	1	4
69	0	1	3	3	2	3	5	3	2	3	3
70	2	6	2	5	6	6	6	4	6	6	2
71	2	1	4	3	4	3	4	1	3	4	4
72	1	2	5	2	3	2	2	5	4	3	4
73	2	6	5	3	3	2	3	4	3	2	2
74	2	3	1	5	6	6	6	4	5	4	4
75	1	2	4	3	3	4	4	1	3	4	4
76	1	2	3	1	3	5	4	2	4	4	4
77	1	2	1	5	5	5	6	4	4	5	2
78	0	1	4	4	4	4	4	1	4	4	4
79	0	1	4	4	4	4	4	4	4	4	4
80	0	1	4	2	3	3	3	1	1	4	5
81	1	5	5	5	2	3	3	4	2	2	6
82	0	1	4	5	6	7	3	4	7	7	2
83	0	1	3	5	4	3	5	5	6	3	3
84	2	9	2	5	1	2	7	2	7	6	1
85	2	7	4	6	5	5	6	4	7	5	3
86	0	2	4	3	3	3	5	4	3	4	5
87											
88	1	1	5	4	3	4	5	4	3	3	5
89	2	3	4	4	3	3	5	4	2	3	5
90	2	7	3	5	5	3	3	4	5	5	2
91	2	3	5	5	3	3	5	4	3	4	5
92	2	8	6	5	2	2	5	6	2	4	5
93	1	3	5	4	3	3	4	4	3	4	4
94	0	2	4	4	5	5	5	4	4	5	4
95	2	6	5	2	2	3	7	6	2	3	5
96	1	3	3	3	3	3	4	4	3	4	4
97	2	8	3	4	3	4	5	4	3	4	4
98	2	5	3	4	4	5	5	4	5	4	4
99	0	6	4	4	4	5	6	4	4	4	5
100	0	1	4	4	4	4	4	4	4	4	4
101	2	5	2	6	6	5	5	4	6	5	3
103	1	5	3	5	5	4	3	4	5	5	3
104	1	6	6	2	2	2	3	6	2	3	2
105	1	8	7	1	1	1	2	4	1	1	1
106	2	3	5	2	2	3	3	5	3	2	4
107	2	4	2	3	3	4	4	4	4	4	4
108	2	6	4	5	4	5	6	5	5	3	3
109	0	3	3	3	3	3	3	3	3	3	3
110	1	4	3	5	5	4	4	4	5	3	3
111	2	4	4	4	6	5	7	5	5	4	4
112	0	1	4	4	4	5	5	5	5	4	4
113	0	6	5	4	5	4	3	4	5	4	4
114	2	8	3	5	6	4	6	6	5	4	3

115	0	1	4	3	3	3	4	3	2	4	5
116	0	3	3	3	5	4	6	5	5	4	4
117	1	6	6	4	2	2	6	6	2	4	5
118	1	8	5	3	4	5	5	4	4	3	4
119	0	4	3	4	4	4	5	2	3	3	3
120	1	4	4	1	1	2	4	4	2	2	2
121	0	3	4	4	4	4	4	4	5	5	4
122	2	5	4	2	2	4	4	4	1	1	2
123	0	3	4	5	4	4	5	4	3	4	4
124	2	2	3	4	4	3	4	4	4	4	4
125	2	2	5	3	3	3	5	4	2	3	5
126	1	2	4	3	4	5	6	5	3	4	5
127	0	4	2	5	5	5	5	4	5	5	2
128	2	5	4	3	3	3	4	3	3	3	3
129	0	1	5	3	3	3	3	4	4	4	4
130	2	9	6	1	2	2	5	4	1	4	6
131	2	1	4	3	3	4	5	4	3	4	6
132	2	4	4	4	4	4	3	4	5	4	5
133	2	7	4	3	5	4	3	4	4	4	5
134	1	3	5	1	2	2	3	3	1	3	6
135	2	7	4	5	3	5	5	4	4	4	3
136	2	6	3	5	5	5	4	2	5	5	2
137	2	7	5	4	3	3	5	4	3	4	4
138	2	5	5	3	3	3	4	4	3	4	4
139	0	1	4	3	3	5	3	4	5	6	5
140	2	9	7	2	1	2	6	4	2	3	6
141	2	9	2	6	6	6	4	4	6	4	2
142	2	3	3	4	5	5	5	4	5	4	4
143	0	1	3	4	3	6	5	5	5	5	3
144	2	6	4	3	3	3	2	4	2	4	3
145	0	1	2	5	5	5	5	4	5	5	2
146	1	1	4	4	5	4	4	5	5	5	4
147	0	2	5	3	4	4	6	1	5	5	4
148	2	1	5	2	2	2	2	5	2	2	5
149	2	1	4	4	4	4	4	4	4	4	4
150	1	1	1	4	4	4	5	1	5	4	1
151	0	2	3	5	6	5	5	5	6	4	3
152	1	3	3	5	5	3	5	6	6	6	2
153	2	6	7	1	1	2	6	1	1	1	6
154	0	2	2	5	5	5	6	4	5	5	2
155	0	1	4	4	5	4	4	5	5	3	4
156	2	2	6	4	2	2	2	2	2	4	6
157	1	1	1	6	6	6	6	1	7	4	1
158	2	3	3	4	5	5	6	3	5	5	2
159	0	1	4	4	4	4	4	1	4	4	4
160	2	6	2	4	5	6	5	4	6	5	2
161	1	3	4	4	4	4	4	4	3	4	4
162	2	8	2	2	3	5	4	5	7	4	5
163	0	1	4	4	4	4	4	4	4	4	4
164	2	3	3	4	5	5	5	1	4	5	2
165	0	5	4	4	4	5	4	4	3	4	4
166	2	6	3	5	6	5	5	3	5	6	3
167	1	2	5	6	5	3	6	6	6	5	2
168	0	3	4	5	3	3	3	4	4	4	4
169	0	1	3	5	6	6	6	5	6	6	3
170	0	1	4	4	5	5	4	4	5	4	4
171	0	1	1	1	6	3	5	2	6	3	1
172	0	1	4	3	3	4	4	4	3	4	5

173	0	3	2	4	3	5	3	4	2	4	2
174	0	1	2	1	4	4	4	4	5	5	2
175	1	1	4	4	4	4	4	4	4	4	4
176	0	1	4	4	5	4	3	4	5	4	4
177	0	1	3	4	3	3	3	4	4	4	4
178	0	2	4	3	3	3	4	4	2	2	5
179	2	3	2	5	6	5	5	3	5	3	2
180	2	1	4	3	4	4	3	4	3	4	4
181	1	1	2	6	6	5	5	4	5	6	4
182	1	2	4	4	5	5	5	4	4	4	4
183	1	4	3	3	3	3	5	3	5	3	3
184	0	1	4	4	5	4	4	4	5	4	4
185	2	4	5	3	3	3	5	4	3	4	4
186	2	5	1	5	5	4	4	4	5	4	4
187	1	1	4	4	4	4	4	4	4	4	4
188	0	3	2	2	4	4	4	3	5	3	3
189	1	1	3	3	3	3	3	3	3	3	3
190	1	3	4	4	4	4	5	4	5	4	4
191	2	2	2	5	6	5	5	4	5	5	3
192	2	5	5	5	5	5	4	4	6	4	4
193	2	4	2	4	3	3	5	2	4	5	2
194	2	6	4	3	4	4	3	3	3	4	4
195	1	4	3	4	3	3	5	5	2	2	6
196	0	2	5	2	2	2	3	3	2	2	5
197	2	1	2	2	2	2	2	2	2	2	2
198	0	1	4	4	4	4	4	4	4	4	4
199	1	2	4	4	4	4	4	4	4	4	4
200	1	4	5	2	2	2	2	2	2	2	6
201	2	3	5	5	6	6	6	5	6	6	4
202	0	1	1	6	7	7	3	3	7	7	1
203	2	1	4	4	4	4	5	4	5	5	4
204	2	5	3	2	3	3	4	3	2	4	5
205	2	5	4	3	6	5	6	4	2	4	6
206	1	5	4	4	4	5	5	4	4	4	4
207	2	4	2	6	4	5	7	4	4	3	3
208	2	7	3	4	5	5	3	4	5	5	3
209	0	2	5	4	3	4	5	4	4	4	4
210	1	2	4	4	4	4	4	4	4	4	4
211	0	1	5	4	3	4	4	4	4	4	4
212	2	7	5	4	4	5	5	4	4	4	4
213	1	1	4	4	3	5	4	4	4	4	4
214	0	1	3	4	4	4	4	4	4	4	4
215	0	3	2	5	5	5	3	4	6	6	2
216	0	1	4	4	4	4	4	4	4	4	4
217	0	1	4	4	4	4	4	4	4	4	4
218	0	1	4	4	4	4	4	4	4	4	4
219	1	2	5	4	3	4	6	4	4	4	4
220	0	1	4	4	4	5	3	4	4	4	4
221	0	1	4	4	4	4	4	4	4	4	4
222	0	1	4	4	4	4	4	4	4	4	4
223	1	1	4	4	6	4	2	4	5	4	4
224	0	1	4	4	4	4	4	4	4	4	4
225	0	1	4	4	4	3	4	4	4	4	4
226	0	1	4	5	4	4	4	4	5	4	4
227	1	1	4	4	4	4	5	4	4	4	4
228	0	3	4	4	4	4	4	4	4	4	4
229	0	1	4	4	4	4	4	4	4	4	4
230	0	4	4	4	4	4	5	4	4	4	4

231	0	1	4	4	4	4	4	4	4	4	4
232	0	1									
233	0	2	4	4	4	4	4	4	4	4	4
234	2	4	5	4	5	4	4	4	5	4	4
235	0	2	4	4	4	4	4	4	4	4	4
236	2	1	4	4	4	5	5	4	4	4	4
237	0	1	4	4	4	4	4	4	4	4	4
238	0	1	3	4	4	4	4	4	3	4	4
239	0	1	1	1	2	2	2	2	2	2	2
240	0	1	4	5	5	4	4	5	4	4	4
241	0	2	4	4	4	4	4	4	5	4	4
242	0	1	3	3	3	3	3	3	3	3	3
243	0	2	4	4	5	6	6	4	6	6	2
244	2	1	4	2	4	3	5	2	3	3	5
245	2	3	4	4	4	4	4	4	4	4	4
246	2	4	5	4	3	3	5	3	5	5	4
247	2	5	2	4	5	5	5	4	5	4	4
248	2	6	2	5	6	6	7	6	6	6	2
249	2	2	4	4	4	4	5	4	4	4	4
250	0	1	2	5	5	6	6	4	6	5	2
251	0	1	4	4	4	4	4	4	4	4	4
252	0	1	4	3	4	4	4	4	4	4	4
253	1	1	4	4	5	5	5	4	5	5	4
254	0	2	3	3	5	5	5	3	3	4	4
255	2	5	5	4	3	3	5	4	3	4	3
256	0	1	4	4	5	5	4	4	4	4	4
257	1	2	4	4	4	4	4	4	4	4	4
258	2	1	2	4	3	3	4	4	4	5	2
259	2	2	4	4	4	4	4	4	3	4	4
260	2	7	4	5	5	3	5	6	5	5	3
261	1	1	4	3	3	4	3	4	3	4	4
262	2	2	3	4	5	5	4	4	4	4	4
263	2	1	4	5	4	3	3	5	4	3	4
264	1	1	4	5	5	5	4	4	5	4	3
265	2	9	2	6	6	6	5	2	6	6	5
266	2	9	5	3	3	3	5	3	3	3	4
267	2	9	6	2	3	3	4	4	2	3	5
268	1	3	5	4	4	4	4	4	4	4	4
269	2	6	4	4	3	3	5	4	5	4	4
270	1	5	3	5	5	4	4	2	4	4	3
271	2	2	4	2	3	3	4	3	3	4	3
272	2	8	6	2	2	3	6	3	5	4	2
273	1	6	4	6	6	7	4	4	5	5	3
274	2	3	2	5	5	4	6	4	5	4	3
275	2	5	4	4	4	4	4	4	4	4	4
276	0	1	4	3	4	4	4	4	4	4	4
277	0	1	4	2	3	3	4	4	3	4	4
278	2	8	5	3	3	3	5	4	2	4	3
279	2	6	5	4	3	3	5	4	3	3	3
280	2	3	4	3	3	3	4	3	3	4	4
281	0	1	4	4	4	4	4	4	4	4	4
282	0	2	4	2	2	3	4	4	2	3	5
283	0	2	3	3	3	3	3	4	3	5	3
284	0	1	4	3	3	3	3	3	3	3	3
285	0	2	3	4	4	4	6	3	3	3	3
286	1	2	4	4	4	4	4	4	4	4	4
287	0	1	4	3	4	4	4	3	3	4	4
288	2	4	2	6	5	6	5	4	6	5	2

289	2	1	4	4	4	4	4	4	4	4	4
290	0	1	4	4	4	4	4	4	4	4	4
291	2	1	4	6	5	4	5	4	5	4	4
292	2	1	7	1	4	2	4	4	1	3	4
293	2	3	4	5	5	6	4	4	5	6	4
294	1	1	3	4	4	5	5	1	3	4	4
295	0	1	4	4	4	4	4	4	4	4	4
296	2	3	4	4	5	5	4	4	5	4	2
297	2	4	4	5	5	4	4	4	4	4	4
298	1	5	4	4	4	3	4	4	4	4	4
299	0	1	4	4	4	4	4	4	4	4	4
300	0	1	3	3	3	3	3	3	3	3	3
301	1	2	5	4	4	3	4	5	3	4	3
302	0	1	4	4	5	5	5	4	5	4	4
303	1	4	3	2	2	3	3	4	2	3	4
304	1	1	2	3	6	6	2	3	6	4	3
305	2	1	4	4	4	4	4	4	4	4	4
306	1	1	4	4	4	3	5	4	4	4	4
307	2	7	2	6	6	5	5	4	6	5	2
308	2	5	4	4	4	5	5	4	5	4	4
309	0	1	5	4	3	4	4	4	3	4	4
310	1	4	3	4	4	3	5	4	4	4	3
311	2	7	2	5	6	7	6	4	5	4	2
312	1	1	4	4	4	4	4	4	4	4	4
313	0	2	3	3	3	4	5	3	3	3	3
314	1	1	4	4	4	4	4	4	4	4	4
315	2	8	2	5	6	6	4	2	6	4	2
316	2	7	3	6	3	3	4	5	4	3	3
317	0	1	3	2	4	4	4	4	4	4	4
318	2	2	4	4	3	4	4	4	3	4	4
319	2	1	6	5	3	3	6	4	5	5	3
320	2	2	4	4	4	4	4	4	4	4	4
321	0	1	4	3	2	3	3	4	3	4	4
322	2	2	4	4	4	4	4	4	4	4	4
323	2	6	5	2	3	2	2	1	2	4	3
324	0	1	4	4	4	4	5	4	5	4	4
325	1	1	1	4	6	3	2	4	5	4	3
326	0	2	3	5	3	6	5	2	5	5	2
327	2	4	4	5	5	4	4	5	5	4	4
328	1	2	4	3	4	3	3	2	2	2	3
329	0	2	4	5	4	4	4	4	5	4	5
330	1	4	5	5	5	3	3	5	3	3	5
331	2	6	5	3	3	3	5	3	3	3	5
332	2	6	6	3	3	3	3	5	3	3	4
333	2	8	4	6	4	4	6	4	5	4	6
334	0	1	1	5	5	5	2	4	5	5	1
335	2	6	3	3	3	4	4	3	3	3	5
336	2	2	5	7	5	5	7	6	7	4	5
337	0	2	4	4	4	4	4	4	4	4	4
338	1	2	5	2	4	3	3	2	2	3	3
339	2	5	6	6	5	5	5	6	5	5	5
340	1	2	5	3	4	5	5	3	4	4	3
341	0	1	3	3	3	3	3	3	3	3	3
342	0	2	4	4	5	4	6	4	4	4	2
343	2	2	4	4	4	5	5	1	4	4	3
344	2	4	5	1	2	1	3	4	2	2	6
345	0	1	4	4	3	3	4	4	3	3	4
346	0	1	3	2	4	3	4	4	1	3	4

347	0	1	3	3	3	3	4	4	4	4	4
348	1	2	4	4	5	5	5	4	5	5	3
349	2	7	3	6	4	4	5	6	6	4	3
350	1	7	6	6	5	3	3	4	6	3	4
351	0	1	2	4	6	5	5	5	4	6	4
352	2	6	6	1	1	2	6	4	2	2	6
353	0	1	6	1	1	1	5	1	1	1	6
354	0	1	3	4	6	4	6	4	4	5	3
355	2	8	2	6	5	4	4	4	5	4	3
356	2	2	4	4	4	4	3	4	4	6	4
357	2	7	5	4	3	3	6	4	3	4	5
358	2	1	6	4	3	2	5	4	2	3	6
359	0	1	4	5	4	4	5	5	5	4	3
360	2	7	5	2	2	2	6	1	1	4	3
361	2	2	3	3	5	5	4	4	5	4	4
362	2	1	5	4	5	4	5	4	4	4	5
363	0	1	4	4	5	5	4	4	4	4	4
364	1	1	5	3	3	3	3	1	1	1	1
365	0	2	5	3	3	3	5	2	3	4	5
366	2	7	5	3	3	3	3	3	2	3	5
367	2	3	5	3	5	5	4	4	3	4	5
368	2	8	7	3	4	3	6	2	2	2	5
369	2	1	4	3	4	4	3	4	3	3	5
370	2	5	4	3	4	4	5	3	3	3	2
371	1	4	1	6	7	7	6	2	7	6	2
372	2	5	5	2	4	3	3	2	3	3	4
373	2	4	6	5	6	6	5	5	5	5	5
374	0	1	5	2	2	4	3	4	3	4	4
375	2	2	4	4	3	4	4	4	3	4	4
376	0	1	7	1	1	1	4	1	1	1	7
377	2	1	3	3	3	3	2	3	3	3	2
378	0	2	4	5	5	4	4	4	5	4	4
379	1	1	4	4	4	4	4	4	4	4	4
380	2	6	3	4	4	3	4	4	4	3	4
381	2	5	4	3	4	4	4	4	3	3	4
382	0	1	3	2	2	2	2	4	2	2	4
383	0	1	5	2	3	3	4	4	2	4	3
384	0	1	4	4	4	4	5	4	4	4	4
385	2	5	5	5	3	3	3	5	3	3	5
386	2	5	3	4	5	4	4	5	5	5	3
387	2	9	5	6	7	5	5	4	5	5	1
388	2	2	3	3	4	4	4	5	4	4	4
389	0	2	5	5	4	4	4	4	4	1	4
390	2	7	5	4	4	4	4	5	4	4	5
391	0	6	5	3	4	3	3	4	3	4	4
392	2	6	5	3	3	3	3	4	2	2	6
393	1	2	5	2	2	3	4	3	2	2	4
394	2	1	3	4	5	5	5	4	5	4	3
395	0	1	4	4	3	3	3	3	2	3	4
