# Supplementary material for: An Exploratory Study on the Regulatory Effect of Autonomous Sensory Meridian Response on Anxiety: Evidence From Functional Near‐Infrared Brain Imaging Technology
Source: Eur J Neurosci. 2025 Sep 14;62(5):e70251. doi: 10.1111/ejn.70251 (PMC12434388; doi:10.1111/ejn.70251)
Supplement: Supplementary file 2 — Appendix S2: Supporting information. [file EJN-62-0-s004.pdf]

The IAT - Anxiety Paradigm

	Block1	Block2	Block3	Block4	Block5
Description	Target discrimination	Attribute discrimination	Initial combined task	Reversed combined task	Reversed combined task
Category labels	● Me<br>Others ●	● Anxiety<br>Calmness ●	● Me<br>● Anxiety<br>Others ●<br>Calmness ●	● Calmness<br>Anxiety ●	● Me<br>● Calmness<br>Others ●<br>Anxiety ●
Sample items	○ 我们<br>○ 本人<br>○ 自身<br>他们 ○<br>你们 ○<br>旁人 ○	○ 焦虑的<br>○ 忧虑的<br>○ 烦恼的<br>平静的 ○<br>自然的 ○<br>安心的 ○	○ 我们<br>○ 焦虑的<br>他们 ○<br>平静的 ○	○ 平静的<br>○ 自然的<br>○ 安心的<br>焦虑的 ○<br>忧虑的 ○<br>烦恼的 ○	○ 我们<br>○ 平静的<br>他们 ○<br>忧虑的 ○
Trials	20	20	20+60	20	20+60
