# Supplementary material for: An Exploratory Study on the Regulatory Effect of Autonomous Sensory Meridian Response on Anxiety: Evidence From Functional Near‐Infrared Brain Imaging Technology
Source: Eur J Neurosci. 2025 Sep 14;62(5):e70251. doi: 10.1111/ejn.70251 (PMC12434388; doi:10.1111/ejn.70251)
Supplement: Supplementary file 3 — Appendix S3: Supporting information. [file EJN-62-0-s007.pdf]

Video information

Num	Mov	Title	Sex	Dialogue	Intensity	Usage Phase	Screenshot
1	53. mp4	whispering	female	yes	middle	screening experiment	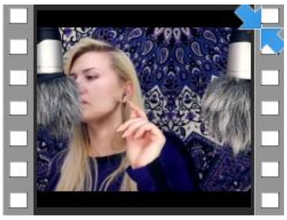
2	54.mp 4	whispering and personal attention	female	yes	low	1.screening experiment<br>2.the anxiety relief phase of the formal experiment	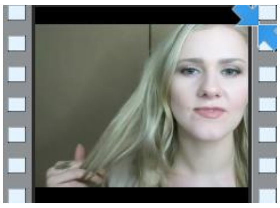
