# Supplementary figures and images for: An Exploratory Study on the Regulatory Effect of Autonomous Sensory Meridian Response on Anxiety: Evidence From Functional Near‐Infrared Brain Imaging Technology
Source: Eur J Neurosci. 2025 Sep 14;62(5):e70251. doi: 10.1111/ejn.70251 (PMC12434388; doi:10.1111/ejn.70251)

The position of channels

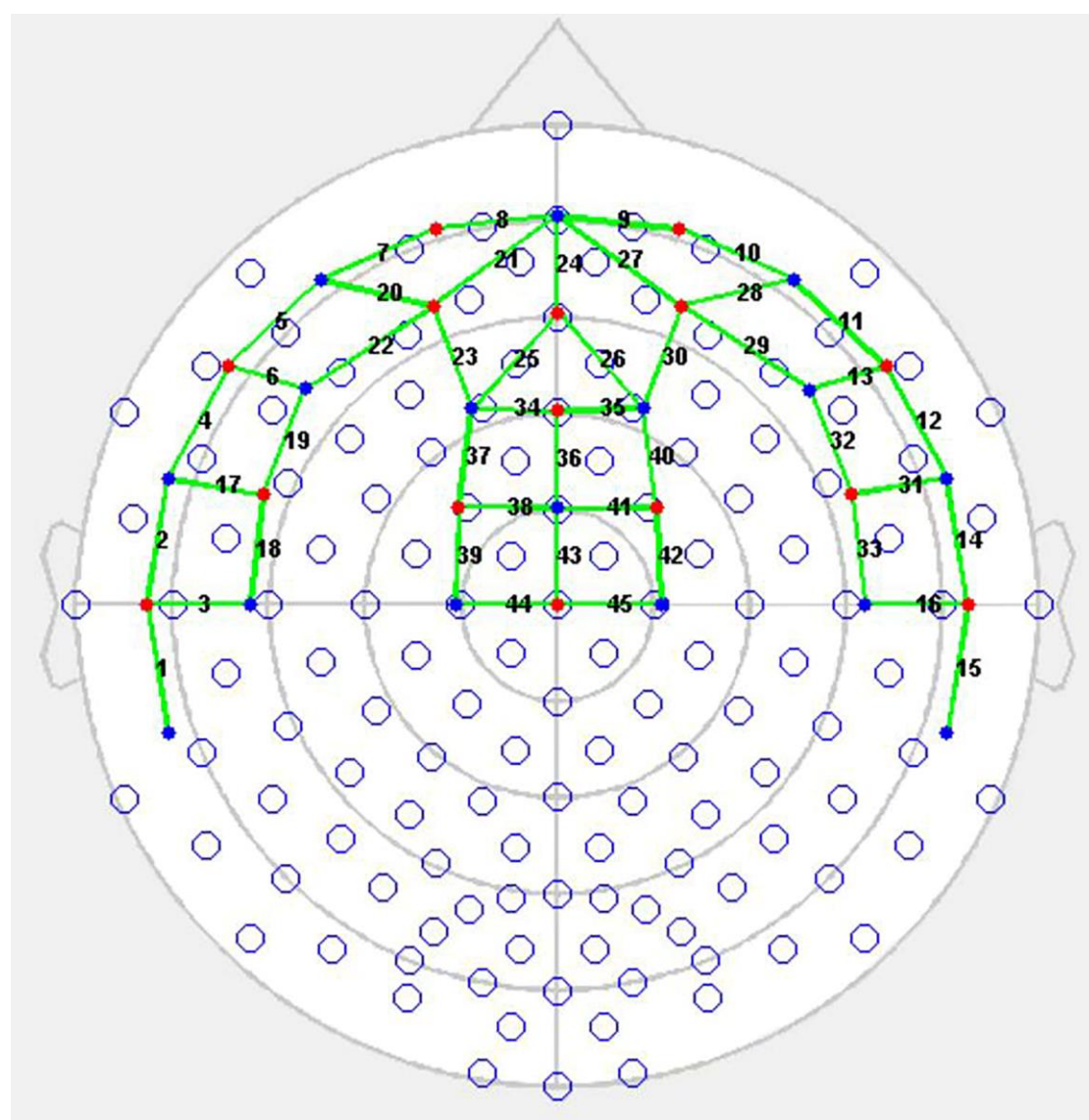

Supplement: Supplementary file 4 — Appendix S4: Supporting information. [file EJN-62-0-s005.pdf]
