# Supplementary material for: An Exploratory Study on the Regulatory Effect of Autonomous Sensory Meridian Response on Anxiety: Evidence From Functional Near‐Infrared Brain Imaging Technology
Source: Eur J Neurosci. 2025 Sep 14;62(5):e70251. doi: 10.1111/ejn.70251 (PMC12434388; doi:10.1111/ejn.70251)
Supplement: Supplementary file 5 — Appendix S5: Supporting information. [file EJN-62-0-s002.pdf]

## fNIRS data

With the fNIRS signal of 785 and 830 nm wavelengths, we used Nirslab to set related events and employed “discontinuities” and “spike artifacts” to remove the motion artifacts caused by head movement and electrode displacement. We used a high cut-off frequency (0.2 Hz) and a low cut-off frequency (0.01 Hz) to perform band-pass filtering to eliminate the influence of noises from instrumentation and drift, as well as others. Then we applied the modified Beer-Lambert law to extract the oxy-Hb and deoxy-Hb signals.

In the Data Viewer module, the time series of oxy-Hb and deoxy-Hb, changing with related events viewed through the Plot chart, carries the functional near-infrared spectrum signals of oxy-Hb (red line) and deoxy-Hb (blue line). We utilized the hemodynamic data to view the brain activation area map of the event through the map chart.

In the Data Analysis module, we used statistical parametric mapping software to examine the segmented fNIRS signals separately. Although both oxy-Hb and deoxy-Hb reflect cerebral blood flow in the fNIRS, oxy-Hb is more sensitive than deoxy-Hb (Zhang et al., 2017). Therefore, only oxy-Hb is further analyzed in this study. We computed the average value of oxy-Hb changes in 0-back and 3-back and then input the data into the general linear model to obtain a beta value for oxy-Hb, assuming that the hemodynamic response function peaks at 5 seconds (Boynton et al., 2012), and the differences between 3-back and 0-back, ASMR and 3-back, ASMR and non-ASMR, and sensation and resting state were compared between high and low trait anxiety groups by paired sample t-test. The resting state is defined as a period of steady breathing by the participant prior to the formal experiment.
