# Supplementary material for: An Exploratory Study on the Regulatory Effect of Autonomous Sensory Meridian Response on Anxiety: Evidence From Functional Near‐Infrared Brain Imaging Technology
Source: Eur J Neurosci. 2025 Sep 14;62(5):e70251. doi: 10.1111/ejn.70251 (PMC12434388; doi:10.1111/ejn.70251)
Supplement: Supplementary file 7 — Appendix S7: Supporting information. [file EJN-62-0-s001.pdf]

Detailed experimental data

sub1	sub2	d(IAT-anxiety)	duration	ety(1-low	age	ss(1-right	sex(1-man;2-women)
123	14	0.274449674	2	2	18	1	1
121	21	0.675484889	5	2	22	1	2
120	19	0.347775581	1	2	20	1	2
119	33	0.999002872	4	2	21	1	1
118	43	0.823583458	7	2	18	1	1
117	4	0.823149075	4	2	19	1	1
116	31	0.70503904	5	2	19	1	2
114	38	0.576032177	1	2	20	1	1
113	13	0.510517641	2	2	19	1	2
112	28	0.48401839	7	2	20	1	2
111	39	0.425969119	5	2	19	1	2
109	12	0.408552667	7	2	20	1	2
108	41	0.386282853	2	2	18	1	2
7	32	0.384653559	7	2	18	1	2
36	10	0.337809546	9	2	19	1	2
18	35	0.253581209	4	2	19	1	2
103	37	0.225694076	3	2	20	1	2
102	40	0.195837428	1	2	18	1	2
101	16	0.192690048	6	2	20	1	2
42	34	-0.904588826	5	1	19	1	2
41	20	-0.762378066	7	1	22	1	2
39	2	-1.02074857	3	1	20	1	2
36	5	-0.653515839	4	1	19	1	2
35	18	-0.656902835	4	1	20	1	2
34	17	-0.904569046	4	1	19	1	1
32	7	-1.243017193	4	1	19	1	2
30	30	-0.701358322	5	1	19	1	1
28	6	-0.849771514	5	1	20	1	1
23	1	-0.723347789	6	1	18	1	2
21	27	-0.85474379	6	1	17	1	1
19	29	-0.895880483	6	1	18	1	2
15	22	-0.638399337	7	1	18	1	2
14	8	-0.692762306	7	1	19	1	2
13	11	-0.722068795	7	1	19	1	1
12	23	-0.86773372	7	1	18	1	1
11	15	-0.969941303	7	1	18	1	2
10	36	-1.526856234	7	1	17	1	2
9	42	-0.642052491	8	1	19	1	2

sub1	sub2	zeroacc	zerort	oneacc	onert	twoacc	twort	zeroacc	zerort
123	14	1	3.67	0.92	3.81	0.83	3.94	1	3.67
121	21	1	3.74	0.94	3.82	0.86	3.37	1	3.74
120	19	1	3.66	0.94	3.74	0.89	3.44	1	3.66
119	33	1	3.71	0.94	3.85	0.72	4.76	1	3.71
118	43	1	3.6	0.94	3.74	0.94	3.37	1	3.6
117	4	1	3.84	0.94	3.92	0.83	3.41	1	3.84
116	31	1	3.61	0.92	3.88	1	3.52	1	3.61
114	38	1	3.6	0.94	3.76	1	3.61	1	3.6
113	13	1	3.67	0.92	3.96	0.67	3.52	1	3.67
112	28	1	3.63	0.94	3.78	1	3.81	1	3.63
111	39	1	3.7	0.94	3.84	1	3.53	1	3.7
109	12	1	3.68	0.94	3.79	0.72	4.75	1	3.68
108	41	0.94	3.99	0.86	4.01	0.78	4.42	0.94	3.99
7	32	1	3.59	0.94	3.73	0.72	4.74	1	3.59
36	10	1	3.6	0.94	3.73	1	3.5	1	3.6
18	35	1	3.65	0.94	3.8	0.69	4.63	1	3.65
103	37	0.97	3.51	0.92	3.68	0.78	4.34	0.97	3.51
102	40	1	3.6	0.94	3.74	0.72	4.74	1	3.6
101	16	1	3.67	0.94	3.77	0.92	3.4	1	3.67
42	34	1	3.63	0.93	3.79	0.83	4.09	1	3.63
41	20	1	3.63	0.94	3.74	0.94	3.29	1	3.63
39	2	1	3.59	0.92	3.78	0.89	3.28	1	3.59
36	5	1	3.61	0.94	3.74	0.94	3.93	1	3.61
35	18	1	3.63	0.92	3.87	0.53	4.27	1	3.63
34	17	1	3.62	0.94	3.8	0.97	3.86	1	3.62
32	7	1	3.6	0.94	3.74	0.92	3.48	1	3.6
30	30	1	3.64	0.94	3.79	1	3.85	1	3.64
28	6	0.94	3.7	0.94	3.78	0.83	4.45	0.94	3.7
23	1	1	3.63	0.94	3.75	0.69	4.62	1	3.63
21	27	1	3.61	0.94	3.73	0.78	4.49	1	3.61
19	29	1	3.62	0.94	3.74	0.72	4.74	1	3.62
15	22	1	3.65	0.94	3.79	0.72	4.76	1	3.65
14	8	1	3.62	0.94	3.75	0.69	4.87	1	3.62
13	11	1	3.62	0.94	3.75	0.72	4.75	1	3.62
12	23	1	3.6	0.94	3.75	1	3.59	1	3.6
11	15	1	3.74	0.94	3.85	0.72	4.76	1	3.74
10	36	1	3.65	0.94	3.74	1	3.54	1	3.65
9	42	1	3.64	0.94	3.76	0.89	3.68	1	3.64
